# Supplementary material for: Effects of combined nitrification inhibitors on soil nitrification, maize yield and nitrogen use efficiency in three agricultural soils
Source: PLoS One. 2022 Aug 22;17(8):e0272935. doi: 10.1371/journal.pone.0272935 (PMC9394818; doi:10.1371/journal.pone.0272935)
Supplement: S1 Table — (DOCX) [file pone.0272935.s001.docx]

| Soil type | Treatment | Nitrification inhibition rate (%) | | | |
| --- | --- | --- | --- | --- | --- |
|  |  | Seedling | Elongation | Filling | Maturity |
| Cinnamon soil | AD | 0.00±0.00Cb | 19.91±4.58Aa | 18.66±6.49Aa | 0.00±0.00Bb |
|  | AN | 9.59±0.74Ba | 15.84±0.10Ca | 14.98±3.94Ba | 0.00±0.00Bb |
|  | ADN | 0.00±0.00Cb | 0.00±0.00Bb | 0.00±0.00Bb | 13.08±3.78Aa |
| Brown soil | AD | 11.77±1.98Bc | 0.00±0.00Bc | 0.00±0.00Cc | 20.79±1.62Aa |
|  | AN | 18.28±1.34Ab | 25.75±2.51Bb | 67.91±3.78Aa | 7.09±2.30Ac |
|  | ADN | 21.69±0.84Aa | 36.61±6.22Aa | 38.05±7.72Bb | 11.55±0.48Ab |
| Red  soil | AD | 19.56±0.65Aa | 22.81±1.43Ab | 0.00±0.00Bb | 0.00±0.00Ba |
|  | AN | 0.00±0.00Cc | 29.12±0.31Aa | 0.00±0.00Cb | 0.00±0.00Ba |
|  | ADN | 4.37±1.07Bb | 0.00±0.00Bc | 52.01±1.50Aa | 0.00±0.00Ba |

Table S1. Nitrification inhibition rate of different treatments in three agricultural soils during four sampling periods.

Treatment: AD, AS + 3, 4-dimethylpyrazole phosphate (DMPP); AN, AS + nitrogen protectant (N-GD); ADN, AS + 3, 4-dimethylpyrazole phosphate (DMPP) + nitrogen protectant (N-GD). Values represent means ± stand error (n = 3). Values within the same column followed by the different capital letters indicated significant differences between different soils in the same treatment at *P* < 0.05 by Duncan test; values within the same column followed by the different lowercase letters indicated significant differences between different treatments in the same soil at *P* < 0.05 by Duncan test.
