# Supplementary material for: Effects of combined nitrification inhibitors on soil nitrification, maize yield and nitrogen use efficiency in three agricultural soils
Source: PLoS One. 2022 Aug 22;17(8):e0272935. doi: 10.1371/journal.pone.0272935 (PMC9394818; doi:10.1371/journal.pone.0272935)

PLOS ONE

Effects of combined nitrification inhibitors on soil nitrification, maize yield and nitrogen use efficiency in three agricultural soils

--Manuscript Draft--

**Manuscript Number:**	PONE-D-21-09541
**Article Type:**	Research Article
**Full Title:**	Effects of combined nitrification inhibitors on soil nitrification, maize yield and nitrogen use efficiency in three agricultural soils
**Short Title:**	Effects of combined nitrification inhibitors on soil nitrification, maize yield and nitrogen use efficiency
**Corresponding Author:**	Dongpo Li<br>Institute of Applied Ecology Chinese Academy of Sciences<br>Liaoning, CHINA
**Keywords:**	nitrification inhibitors; ammonium nitrogen; nitrate nitrogen; yield, nitrogen use efficiency
**Abstract:**	The application of nitrification inhibitors (NIs) with nitrogen (N) fertilizer is one of the most efficiency ways to improve nitrogen use efficiency (NUE). To fully understand the efficiency of NIs with N fertilizer on soil nitrification, yield and NUE of maize (Zea mays L.), we conducted an outdoor pot experiment with different NIs in three soils with different pH. In the present research, five treatments were established: no fertilizer (Control); ammonium sulfate (AS); ammonium sulfate + 3, 4-dimethyl-pyrazolate phosphate (DMPP) (AD); ammonium sulfate + nitrogen protectant (N-GD) (AN); ammonium sulfate + 3, 4-dimethyl-pyrazolate phosphate + nitrogen protectant (ADN). The result showed that NIs treatments (AD, AN and ADN) significantly reduced soil nitrification in the brown and red soil, especially in AD and ADN, which decreased apparent nitrification rate 28% - 44% ($P < 0.05$). All NIs treatments significantly increased yield and NUE of maize in three soils, especially ADN in the cinnamon soil and AD in the red soil were more efficiency, which significantly increased maize yield and apparent nitrogen recovery by 5.07 times and 6.81 times, 4.39 and 8.16 times, respectively. No significant difference on maize yield was found in the brown soil, but AN significantly increased apparent nitrogen recovery by 70%. In conclusion, all NIs treatments performed better in inhibiting nitrification in the brown soil than in the cinnamon and red soil. DMPP and N-GD were more effective in the red soil than in the cinnamon soil. DMPP + N-GD was the most efficient in improving maize yield in the brown soil than other two soils, while DMPP performed better in improving apparent nitrogen recovery in the red soil than other two soils. Soil pH plays an important role in the effectiveness of DMPP, N-GD and their combination in influencing soil nitrification, yield and NUE of maize.
**Order of Authors:**	Lei Cui
	Dongpo Li, Ph.D.
	Zhijie Wu
	Yan Xue
	Furong Xiao
	Ping Gong
	Lili Zhang
	Yuchao Song
	Chunxiao Yu
	Yonghua Li
	Ye Zheng
**Additional Information:**	

Powered by Editorial Manager® and ProduXion Manager® from Aries Systems Corporation

Question	Response
**Financial Disclosure**<br><br>Enter a financial disclosure statement that describes the sources of funding for the work included in this submission. Review the submission guidelines for detailed requirements. View published research articles from *PLOS ONE* for specific examples.<br><br>This statement is required for submission and **will appear in the published article** if the submission is accepted. Please make sure it is accurate.<br><br>**Unfunded studies**<br>Enter: *The author(s) received no specific funding for this work.*<br><br>**Funded studies**<br>Enter a statement with the following details:<br>• Initials of the authors who received each award<br>• Grant numbers awarded to each author<br>• The full name of each funder<br>• URL of each funder website<br>• Did the sponsors or funders play any role in the study design, data collection and analysis, decision to publish, or preparation of the manuscript?<br>• **NO** - Include this sentence at the end of your statement: *The funders had no role in study design, data collection and analysis, decision to publish, or preparation of the manuscript.*<br>• **YES** - Specify the role(s) played.<br><br>* typeset	This work was financially supported by the State Key Program of China (2017YFD0200707), a grant to Dongpo Li, who designed the experiment.We are grateful to the National Field Research Station of Shenyang Agro-ecosystems, Chinese Academy of Sciences, for providing the experimental field.
**Competing Interests**<br><br>Use the instructions below to enter a competing interest statement for this submission. On behalf of all authors, disclose any competing interests that could be perceived to bias this work—acknowledging all financial support and any other relevant financial or non-financial competing interests.	The authors have declared that no competing interests exist.

This statement is required for submission and **will appear in the published article** if the submission is accepted. Please make sure it is accurate and that any funding sources listed in your Funding Information later in the submission form are also declared in your Financial Disclosure statement.<br><br>View published research articles from *PLOS ONE* for specific examples.<br><br>**NO authors have competing interests**<br><br>Enter: *The authors have declared that no competing interests exist.*<br><br>**Authors with competing interests**<br><br>Enter competing interest details beginning with this statement:<br><br>*I have read the journal's policy and the authors of this manuscript have the following competing interests: [insert competing interests here]*<br><br>* typeset	
**Ethics Statement**<br><br>Enter an ethics statement for this submission. This statement is required if the study involved:<br><br>• Human participants<br>• Human specimens or tissue<br>• Vertebrate animals or cephalopods<br>• Vertebrate embryos or tissues<br>• Field research<br><br>Write "N/A" if the submission does not require an ethics statement.<br><br>General guidance is provided below. Consult the submission guidelines for detailed instructions. **Make sure that all information entered here is included in the Methods section of the manuscript.**	N/A

Powered by Editorial Manager® and ProduXion Manager® from Aries Systems Corporation

**Format for specific study types**<br><br>**Human Subject Research (involving human participants and/or tissue)**<br>• Give the name of the institutional review board or ethics committee that approved the study<br>• Include the approval number and/or a statement indicating approval of this research<br>• Indicate the form of consent obtained (written/oral) or the reason that consent was not obtained (e.g. the data were analyzed anonymously)<br><br>**Animal Research (involving vertebrate animals, embryos or tissues)**<br>• Provide the name of the Institutional Animal Care and Use Committee (IACUC) or other relevant ethics board that reviewed the study protocol, and indicate whether they approved this research or granted a formal waiver of ethical approval<br>• Include an approval number if one was obtained<br>• If the study involved *non-human primates*, add *additional details* about animal welfare and steps taken to ameliorate suffering<br>• If anesthesia, euthanasia, or any kind of animal sacrifice is part of the study, include briefly which substances and/or methods were applied<br><br>**Field Research**<br><br>Include the following details if this study involves the collection of plant, animal, or other materials from a natural setting:<br>• Field permit number<br>• Name of the institution or relevant body that granted permission	
**Data Availability**<br><br>Authors are required to make all data underlying the findings described fully available, without restriction, and from the time of publication. PLOS allows rare exceptions to address legal and ethical concerns. See the PLOS Data Policy and FAQ for detailed information.	Yes - all data are fully available without restriction

A Data Availability Statement describing where the data can be found is required at submission. Your answers to this question constitute the Data Availability Statement and **will be published in the article**, if accepted.<br><br>**Important:** Stating 'data available on request from the author' is not sufficient. If your data are only available upon request, select 'No' for the first question and explain your exceptional situation in the text box.<br><br>Do the authors confirm that all data underlying the findings described in their manuscript are fully available without restriction?	
**Describe where the data may be found in full sentences. If you are copying our sample text, replace any instances of XXX with the appropriate details.**<br><br>• If the data are **held or will be held in a public repository**, include URLs, accession numbers or DOIs. If this information will only be available after acceptance, indicate this by ticking the box below. For example: *All XXX files are available from the XXX database (accession number(s) XXX, XXX.*).<br>• If the data are all contained **within the manuscript and/or Supporting Information files**, enter the following: *All relevant data are within the manuscript and its Supporting Information files.*<br>• If neither of these applies but you are able to provide **details of access elsewhere**, with or without limitations, please do so. For example:<br><br>*Data cannot be shared publicly because of [XXX]. Data are available from the XXX Institutional Data Access / Ethics Committee (contact via XXX) for researchers who meet the criteria for access to confidential data.*<br><br>*The data underlying the results presented in the study are available*	All relevant data are within the paper.

from (include the name of the third party and contact information or URL).<br>• This text is appropriate if the data are owned by a third party and authors do not have permission to share the data.<br><br>* typeset	
Additional data availability information:	

Powered by Editorial Manager® and ProduXion Manager® from Aries Systems Corporation

Manuscript Click here to access/download;Manuscript;manuscript.docx

Effects of combined nitrification inhibitors on soil nitrification, maize yield and nitrogen use efficiency in three agricultural soils

Lei Cui[1,2¶], Dongpo Li[1*¶], Zhijie Wu[1], Yan Xue[1], Furong Xiao[1,2], Ping Gong[1], Lili zhang[1], Yuchao Song[1], Chunxiao Yu[1], Yonghua Li[3], Ye Zheng[4]

1 Institute of Applied Ecology, Chinese Academy of Sciences, Shenyang，Liaoning, China

2 University of Chinese Academy of Sciences, Beijing, China

3 North Huajin Chemical Industries Group Corporation, Panjin, Liaoning, China

4 Jinxi Natural Gas Chemical Co. Ltd, Huludao, Liaoning, China

* Corresponding authors

E-mail: lidp@iae.ac.cn

¶These authors contributed equally to this work.

Abstract

The application of nitrification inhibitors (NIs) with nitrogen (N) fertilizer is one of the most efficiency ways to improve nitrogen use efficiency (NUE). To fully understand the efficiency of NIs with N fertilizer on soil nitrification, yield and NUE of maize (*Zea mays* L.), we conducted an outdoor pot experiment with different NIs in three soils with different pH. In the present research, five treatments were established: no fertilizer (Control); ammonium sulfate (AS); ammonium sulfate + 3, 4-dimethyl-pyrazolate phosphate (DMPP) (AD); ammonium sulfate + nitrogen protectant (N-GD) (AN); ammonium sulfate + 3, 4-dimethyl-pyrazolate phosphate + nitrogen protectant (ADN). The result showed that NIs treatments (AD, AN and ADN) significantly reduced soil nitrification in the brown and red soil, especially in AD and ADN, which decreased apparent nitrification rate 28% - 44% ($P < 0.05$). All NIs treatments significantly increased yield and NUE of maize in three soils, especially ADN in the cinnamon soil and AD in the red soil were more efficiency, which significantly increased maize yield and apparent nitrogen recovery by 5.07 times and 6.81 times, 4.39 and 8.16 times, respectively. No significant difference on maize yield was found in the brown soil, but AN significantly increased apparent nitrogen recovery by 70%. In conclusion, all NIs treatments performed better in inhibiting nitrification in the brown soil than in the cinnamon and red soil. DMPP and N-GD were more effective in the red soil than in the cinnamon soil. DMPP + N-GD was the most efficient in improving maize yield in the brown soil than other two soils, while DMPP performed better in improving apparent nitrogen recovery in the red soil than other two soils. Soil pH plays an important role in the effectiveness of DMPP, N-GD and their combination in influencing soil nitrification, yield and NUE of maize.

Introduction

Nitrogen (N) fertilizers can increase food production by almost 50%, which made a significantly contribution in alleviating the global food shortage [1]. Hence, tons of N fertilizers are applied to obtain high yield of grain. However, plants are rarely able to absorb more than 50% of the N fertilizer applied to cropping systems, and most of them are lost through NO_3^- leaching, NH_3 volatilization and N_2O emission excessive application of N fertilizers, which caused many problems, such as economical loss, lower nitrogen use efficiency (NUE) and environment pollution [2]. It is necessary, therefore, to find out an efficient way to improve NUE, increase crop yield and mitigate environmental pollution.

Adding nitrification inhibitors (NIs) into ammonium-based fertilizer is one of the considerably effective technologies that can inhibit nitrification, reduce N loss, thus improving crop yield and NUE in agricultural systems [3-4]. Nitrification is a major process impacting N cycling in the high-production agricultural systems [5]. NIs are compounds that delay the process of nitrification of NH_4^+ to NO_3^- and subsequent by depressing the activities of nitrifiers and denitrifiers in soil [6]. Two NIs, 3,4-dimethylpyrazol phosphate (DMPP) and dicyandiamide (DCD), which are commonly used. Many studies documented that the application of DMPP or DCD with organic fertilizer (cattle slurry, cow urine) and inorganic fertilizer (urea, ammonium chloride, ammonium sulfate) can effectively reduce N loss and improve crop and grass yield and NUE in both agricultural and pasture system [7-11]. The application of DCD with urea significantly improved NUE by 12.9% and 14.6%, where reduced NO_3^- leaching by 58.5 and 35.2% and N_2O emission factor by 83.8 and 72.7% , respectively, in two soils (Huangzongrang and chaotu soils) [3]. Fan et al found that the application of NIs with urea increased ammonium concentrations by 0.3-41.1% and decreased nitrate

concentrations by 6.3-34.4% [12]. But there has been little research on the effect of NIs combined with ammonium sulfate in soils. It has been reported that sulfate (SO_4^-) is possibly reduced to thiosulfate ($S_2O_3^{2-}$), which is a nitrification inhibitor [13]. And the synergistic effect of anions and NIs might improve the inhibition effect of NIs [14]. In addition, DCD is more widely used in some countries especially in New Zealand as it is less volatile, easily souble in water and relatively cheaper than other NIs [15]. While DMPP has lower application of one-tenth of DCD dose, and it also has lower phytotoxicity to the plant [16-17]. DMPP is indiscriminate binding with ammoniaum monooxygenase, while DCD is blocking the electron transport in the cytochromes, which deactivate the enzyme responsible for the first step of nitrification [18]. Our study therefore addresses a combined application of different kinds of NIs to refine the use of NIs in soils.

Different NIs have different effects mainly due to the properties of NIs, soil physicochemical properties (soil organic carbon (SOC), pH), fertilizer types (organic or inorganic), field management [19-20]. It is worth to mention that soil pH is one of the main factors to influence the soil nitrification and the efficiency of NIs. In general, nitrification is rapid in soils of pH $\geqslant$ 6.0, but slower in soils of pH $\leq$ 5.0 [19]. A laboratory incubation experiment indicated that DMPP with ammonium chloride was more effective in the neutral soil (pH: 7.0, 93.5%) than in the alkaline soil (pH: 8.0, 85.1% and acid soil (pH: 4.6, 70.5%). Another laboratory incubation experiment had shown that DMPP with urea maintained higher NH_4^+-N concentrations significantly and lower NO_3^--N concentrations, thus reducing N loss and improving NUE in the brown soil (pH: 6.31) [21]. While a field experiment study reported that DCD and urease inhibitor with urea had no significant difference in NH_4^+-N or NO3⁻-N in an acid soil, but in grain yield significantly higher than those of control treatment, however, there was no significant difference between NIs and without NIs [22]. But another field

experiment showed that DMPP significantly increased the annual crop yield by 6% relative to the urea treatment in an alkaline soil [23]. Most previous studies are focused on the effect of NIs alone [24-26], and the responses of different NIs on yield and NUE are various due to the different soils. Therefore, it is urgent to fully understand the effect of different NIs on soil nitrification, yield and NUE of maize in different soils to provide theoretical basis for choosing better NIs. In addition, nitrogen protectant (N-GD) is a mixture of DCD and DMPP in a certain proportion, this is the first research to study the effect of N-GD in different soils.

Brown soil (Hap-Udic Luvisol in the FAO (Food and Agriculture Organization of the United Nations) WRB (World Reference Base) classification system), cinnamon soil (Hap-Ustic Luvisol in the FAO WRB system) and red soil are the typical agricultural soils with different pH in China. In this study, we conduct an outdoor pot experiment with different types of NIs additions in the above three soils. The objective of this study were : 1) to examine the effect of NIs and their combination with ammonium sulfate on soil nitrification in three soils with different pH; 2) to compare the effect of NIs and their combinations in three soils with different pH; 3) to identify the effect of NIs on maize yield and NUE.

Materials and methods

Study site and soil sampling

An outdoor pot experiment was carried out at the National Field Observation and Research Station of Agroecosystems in Shenyang, Liaoning province (41˚31'N, 123˚24'E), in which Dongdan-6531 spring maize (*Zea mays* L., from May to October, 2018) was planted. The mean annual air temperature is 7-8°C, and the mean annual precipitation is approximately 700 mm. The frost-free period is 147-164 days. The soils used in this study were obtained from three sites: a

brown soil (Hap-Udic Luvisol) at Changtu (42°25'N, 123°28'E), Liaoning Province of Northeast China, a cinnamon soil (Hap-Ustic Luvisol) at Chaoyang City (41°49'N, 122°48'E), Liaoning Province of Northeast China, and a red soil at Yingtan National Agro-ecosystem Field Experiment Station (28°15′N, 116°55′E) of the Chinese Academy of Sciences in Jiangxi Province, China. The sampling sites were planted with maize and regularly fertilized. At each site, surface soil (0–20 cm) was collected, sieved to pass through a 5-mm mesh, and homogenized thoroughly. Soil physicochemical properties are shown in Table1.

Table 1. Physicochemical properties of the three agricultural soils (0-20 cm soil layers).

**Soil property**	**Brown soil**	**Cinnamon soil**	**Red soil**
**pH**	5.30 ± 0.02	7.90 ± 0.08	4.70 ± 0.83
**Total C(g kg^{-1})**	9.24 ± 0.02	12.30 ± 0.20	6.86 ± 0.35
**Total N(g kg^{-1})**	0.98 ± 0.08	1.07 ± 0.03	0.89 ± 0.14
**NH_4^+-N(mg kg^{-1})**	13.83 ± 0.71	16.70 ± 0.13	13.20 ± 0.83
**NO_3^--N(mg kg^{-1})**	10.34 ± 0.25	21.28 ± 0.28	11.53 ± 0.98
**Available P(mg kg^{-1})**	11.73 ± 1.04	61.87 ± 2.89	13.70 ± 1.41
**Available K(mg kg^{-1})**	5.70 ± 0.63	91.32 ± 2.77	131.49 ± 7.13

Values indicate mean ± standard deviations (n = 3). TC, total soil carbon; N, total soil nitrogen; NH_4^+-N, ammonium nitrogen; NO_3^--N, nitrate nitrogen; P, phosphorus; K, potassium.

Experimental design

Five treatments were established: 1) no N fertilizer (control); 2) ammonium sulfate (AS); 3) AS+3, 4-dimethyl-pyrazolate phosphate (DMPP) (AD); 4) AS+ nitrogen protectant (N-GD) (AN); 5) AS+ DMPP+N-GD (ADN). Each treatment had six replications. The fertilizer ammonium sulfate

triple superphosphate and potassium chloride were applied with an application rate of 0.3g N, 0.12 g P_2O_5 and 0.15 g K_2O per kg soil, respectively. The application rates of DMPP and N-GD were 0.5% and 1.5%, respectively on the w/w basis of N, and the application rate of every single NI was reduced by 50% in NIs combinations treatment. All the amendments were basal dressed, and air-dried soil (equivalent to 8kg of oven-dry weight) was thoroughly mixed with the corresponding amendments before added a plastic pot of a 28-cm diameter with a 26 cm-height. Five seeds were sown in each pot, and the seedlings were thinned to one per pot after germination and seedling establishment. Soil moisture content was adjusted daily with deionized water to 60% of the maximum water-holding capacity (WHC) during the maize growth period. DMPP and N-GD were supplied by Zhejiang Chemical Institute, China and Spanish corporation, respectively.

Sampling of soil and plant

Soil samples were collected at the seedling stage, elongation stage, filling stage, and maturity stage of maize, *i.e.* 30, 59, 95 and 135 days after sowing, respectively, with three replicates for each treatment. For each pot, five soil cores were collected by using a soil auger (2.5 cm in diameter), and bulked into a composite sample. The sampling were packed with ice pack and transported to laboratory, and passed through a 2 mm sieve before determining inorganic N (NH_4^+-N and NO_3^--N) and moisture content.

The whole plant was harvested at the mature stage of maize from another three replications, and separated into straw and grain. The plant will be placed in oven, dried to constant weight at 65 ℃ to calculate aboveground biomass of maize, then ground to pass through a 0.25 mm sieve for the analysis of total nitrogen (TN).

Analytical procedures

Soil pH was tested by 1:2.5 soil-water ratio with a pH meter, and TN of maize was determined with an elemental analyzer (Vario EL III, Germany) [27]. Soil available phosphorus (AP) was extracted with 0.5 mol L^{-1} $NaHCO_3$ and analyzed by the molybdenum blue method, soil available potassium (AK) was extracted with 1 mol L^{-1} NH_4OAc and determined by the flame photometer method [28]. The soil NH_4^+-N and NO_3^--N content were measured using a Continuous Flow Analyzer (AA III, Germany) [29].

Calculations and statistical analysis

Apparent nitrification rate (%) was calculated using Eq (1) [26]:

$$\text{Apparent nitrification rate} = NO_3^-\text{-N} / (NH_4^+\text{-N} + NO_3^-\text{-N})*100 \qquad (1)$$

Agronomic nitrogen use efficiency (ANUE, g g^{-1}) and apparent nitrogen recovery (AR, %) were calculated by Eq (2) and Eq (3) :

$$ANUE= (Y - Y_C) / N_F \qquad (2)$$

$$AR= (U - U_C) / N_F*100 \qquad (3)$$

Where: Y, grain yield with N fertilizer; Y_C, grain yield with no fertilizer; N_F, the amount of N fertilizer applied; U, plant nitrogen uptake in the aboveground parts with N fertilizer; N_C uptake, plant nitrogen uptake in the above-ground parts with no fertilizer [30].

A two-way ANOVA was used to analyze the effects of soil types (S), treatments (T) and their interactions (S*T) on grain yield, ANUE and AR at the maturity stage of maize. Multiple comparisons among the treatments were further explained using Duncan test. Differences were considered significant at $P < 0.05$. All statistical analyses were performed using SPSS Version 22.0. Graphs were prepared with Origin 9.0.

Results

NH_4^+-N content and NO_3^--N content

We clearly observed that the application of N fertilizer and NIs significantly increased the soil inorganic N content in all three agricultural soils (Fig 1). However, there was no significant differences in soil NH_4^+-N content of all treatments at the later stages of maize growth, which decreased about 10 mg kg^{-1}, 11 mg $kg^{-1}$and 24 mg kg^{-1} in the brown soil, cinnamon soil and red soil, respectively (Fig 1A, C, E, $P > 0.05$). There was the same pattern between of NO_3^--N content with the NH_4^+-N content, which increased at the seedling stage and gradually declined followed the growth of maize (Fig 1B, D, F). In the brown soil, the NH_4^+-N content in AD was the highest than that in other treatments at the seedling stage (Fig 1A, $P < 0.05$). No significant differences were detected between AN and ADN for NH_4^+-N content (Fig 1A, $P > 0.05$). At the elongation stage, the NH_4^+-N content in AD and AN was higher compared with ADN (Fig 1A). We also found that AN maintained higher NH_4^+-N content until the maturity stage of maize. All treatments with NIs significantly decreased NO_3^--N content at the seedling stage, especially AN was lower in NO_3^--N content at the later stages (Fig 1B).

Fig 1. Dynamic changes of NH_4^+-N and NO_3^--N of different treatments in three soils. Treatment: Control, no fertilizer and nitrification inhibitors; AS, ammonium sulphate; AD, AS + 3, 4-dimethylpyrazole phosphate (DMPP); AN, AS + nitrogen protectant (N-GD); ADN, AS + 3, 4-dimethylpyrazole phosphate (DMPP) + nitrogen protectant (N-GD). Error bars represented standard deviations ($n = 3$).

In the cinnamon soil, the addition of other NIs significantly increased soil inorganic N expect for AN. AD maintained higher NH_4^+-N content for a long time, while ADN had higher NO_3^--N content during the growth of maize (Fig 1C, $P < 0.05$). In addition, ADN had higher inorganic

nitrogen than AN (Fig 1D).

In the red soil, all treatments with NIs had significantly increased NH_4^+-N content compared with AS at the seedling stage (Fig 1E, *P* < 0.05). In addition, ADN can maintain higher NH_4^+-N content for a long period. Lower NO_3^- -N values in AD and ADN were found compared with AS, while ADN had no significantly difference in NO_3^- -N content with AS (Fig 1F, *P* >0.05).

Apparent nitrification rate

Apparent nitrification rate (ANR) indicates that intensify of soil nitrification. Apparent nitrification rate was higher in the cinnamon soil than that in the other two soils (Fig 2). In the brown soil, all treatments with NIs had significantly lower ANR over AS during the whole stage of maize growth. AD was the lowest at the seedling stage, followed by AN (Fig 2A). In the cinnamon soil, higher ANR was observed in treatments of adding NIs into N fertilizer, especially in ADN, which showed that NIs significantly increased soil inorganic nitrogen (Fig 2B). In the red soil, the lowest values were observed in both AD and ADN (Fig 2C, *P* < 0.05). In addition, ADN had significantly decreased nitrification during the whole growth stage of maize (Fig 2C).

Fig 2. Changes of apparent nitrification rate of in three soils during four sampling period. Treatment: AS, ammonium sulphate; AD, AS + 3, 4-dimethylpyrazole phosphate (DMPP); AN, AS + nitrogen protectant (N-GD); ADN, AS + 3, 4-dimethylpyrazole phosphate (DMPP) + nitrogen protectant (N-GD). Error bars represented standard deviations (*n* = 3).

Aboveground biomass and nitrogen uptake of maize

Aboveground biomass

In general, NIs with ammonium sulfate significantly increased aboveground biomass compared to AS in all the tested soils (Fig 3, *P* < 0.05). Grain yield of maize was significantly

affected by both soil types and NIs (Table 2, $P < 0.01$). Both grain and straw biomass had the highest value in the brown soil than those in the cinnamon soil and red soil (Fig 3). In the brown soil, grain yield was the highest in AN followed by AD and AN, which were significantly higher than AS (Fig 3A, $P < 0.05$). No significant differences was detected in the application of NIs with ammonium sulfate (Fig 3A, $P > 0.05$).

Fig 3. Aboveground biomass of maize under different treatments in three soils. Treatment: AS, ammonium sulphate; AD, A S+ 3, 4-dimethylpyrazole phosphate (DMPP); AN, AS + nitrogen protectant (N-GD); ADN, AS + 3, 4-dimethylpyrazole phosphate (DMPP) + nitrogen protectant (N-GD). Error bars represented standard deviations ($n = 3$). Different letters indicate significant differences between different treatments at $P < 0.05$ by Duncan test.

Table 2. Two-way ANOVA ($P < 0.05$) of nitrification inhibitor (NI) and soil types (ST) on grain yield, agronomic nitrogen use efficiency (ANUE) and apparent nitrogen recovery (AR) of maize

**Factors**	**DF**	**Grain yield**			**ANUE**			**AR**		
		**SS**	**F**	**p**	**SS**	**F**	**p**	**SS**	**F**	**p**
**NI**	3	12958.1	76.5	***	2250.0	76.5	***	7682.6	127.8	***
**ST**	2	22787.7	201.8	***	3955.7	201.8	***	1274.2	31.8	***
**NI*ST**	6	32174.4	95.0	***	5585.7	95.0	***	13024.2	108.4	***
**Model**	11	67920.2	109.3	***	11791.4	109.3	***	21981.0	99.8	***
**Error**	24	1355.4			235.3			480.8		

SS, the sum of squares;

F value, the ratio of mean squares of two independents samples;

*** Indicates significance at $P < 0.001$.

In the cinnamon soil, the highest value of grain yield was found in ADN than that in other treatments (Fig 3B, $P < 0.05$). AN resulted in significant increase in grain yield when compared to AS ($P < 0.05$), while there was no significant difference between AD and AS for grain yield of maize ($P > 0.05$).

In the red soil, the grain yield in NIs treatments was higher compared with AS (Fig 3C, $P < 0.05$). The highest value was found in AD in comparison to AN and ADN (Fig 3C).

Nitrogen uptake of maize

In the three agricultural soils, all treatments with NIs increased N uptake by maize significantly compared with control (Fig 4, $P < 0.05$). The highest value of grain and total nitrogen uptake of maize was found in the cinnamon soil (Fig 4). In the brown soil, there was no significant difference between AD and AN, which had significantly higher in N uptake of maize than other treatments (Fig 4A, $P < 0.05$). N uptake of maize from ADN was no significantly different from AS (Fig 4A, $P > 0.05$).

Fig 4. Nitrogen uptake of maize under different treatments in three soils. Treatment: AS, ammonium sulphate; AD, AS + 3, 4-dimethylpyrazole phosphate (DMPP); AN, AS + nitrogen protectant (N-GD); ADN, AS + 3, 4-dimethylpyrazole phosphate (DMPP) + nitrogen protectant (N-GD). Error bars represented standard deviations ($n = 3$). Different letters indicate significant differences between different treatments at $P < 0.05$ by Duncan test.

In the cinnamon soil, the highest N uptake of maize values were detected in ADN, which significantly higher than other treatments (Fig 4B, $P < 0.05$). AN had higher N uptake of maize than that of AS, while AD had no significant differences from AS (Fig 4B, $P < 0.05$).

In the red soil, NIs with N fertilizer had significantly increased N uptake of maize compared with AS. AD significantly increased N uptake of maize compared with other treatments (Fig 4C, $P < 0.05$), while ADN was better than AN (Fig 4C).

Agronomy nitrogen use efficiency and apparent nitrogen recovery

Agronomy nitrogen use efficiency (ANUE) and apparent nitrogen recovery (AR) are different aspects to explain the utilization of nitrogen fertilizer in maize. Soil types and NIs significantly affected ANUE and AR (Table 2, $P < 0.05$). ANUE was the highest in the brown soil, while the highest value of AR was found in the red soil (Fig 5). In the brown soil, all treatments with NIs significantly improved ANUE and AR than those of AS (Fig 5A, $P < 0.05$), while there were no significant difference among them in ANUE. AR in AD and AN was significantly higher compared with ADN and AS. No significant differences in AR were detected between ADN and AS (Fig 5A, $P > 0.05$).

Fig 5. Nitrogen use efficiency (ANUE and AR) under different treatments in three soils. ANUE, agronomy nitrogen use efficiency; AR: apparent nitrogen recovery. Treatment: AS, ammonium sulphate; AD, AS + 3, 4-dimethylpyrazole phosphate (DMPP); AN, AS + nitrogen protectant (N-GD); ADN, AS + 3, 4-dimethylpyrazole phosphate (DMPP) + nitrogen protectant (N-GD). Error bars represented standard deviations ($n = 3$). Different letters indicate significant differences between different treatments at $P < 0.05$ by Duncan test.

In the cinnamon soil, the highest values in both ANUE and AR were from ADN, which had significantly different from other treatments. AN was higher in NUE than AD and AS. AD in AR was significantly higher than AS, while ANUE from AD had no significant differences from AS

treatment (Fig 5B, $P > 0.05$).

In the red soil, AD had the highest value in both ANUE and AR among all treatments, which had significantly different with the other treatments (Fig 5C, $P < 0.05$). No significant differences in ANUE were found between AN and ADN (Fig 5C, $P > 0.05$), while higher values in AR were found in ADN than AN.

Discussion

Effects of NIs on soil nitrification

The application of NIs have the potential to slow soil nitrification, thus increasing NH_4^+-N and decreasing NO_3^--N content, and improving crop yield, aboveground biomass, N uptake and NUE. It is worth to mention that nitrification in red soil becomes stronger, which was according with Lu et al [31], mainly due to the tillage during the decades. In the present study, NIs treatments considerably reduced soil nitrification for all three soils, especially in both brown soil and red soil, a result which was mainly driven by lower pH and fertility. The efficacy of NIs in inhibiting nitrification was reduced because of the rapid hydrolysis of NIs at high soil pH [32].

The effect of NIs varied among different types of soil. NIs significantly increased soil NH_4^+ and NO_3^- content in both soils expect for cinnamon soil, which was consistent with Guo et al [33], who found that DCD with urine had the potential to increase NH_4^+ content and maintain it higher for a long time. However, NIs can reduce ANR in all three soils, which was line in with Gong et al [34], who found that DCD and DMPP with urea significantly suppressed potential nitrification rate. In our study, DMPP significantly inhibited nitrification in all soils. DMPP is indiscriminately binding and interaction with ammonium monooxygenase to inhibit the first rate-limiting step of soil nitrification [35]. In addition, DMPP is a heterocyclic N compound with the advantages of low

mobility, slow biodegradation and persistence [36]. We also found that at the later stage of maize, $NH4^+$-N content was gradually declined, which was in line with Zaman et al [12].The main reasons were the decomposition of NIs, N uptake of maize, ammonia volatilization and microbial immobilization [37-38].

Effects of NIs on maize yield and NUE

There are many studies to test the effect of NIs on crops production and N uptake, but it is difficult to draw general conclusions because the performance of the options varied across sites due to soil type (pH, SOM), NIs type, the forms of N fertilizer (organic N fertilizer (urine), inorganic N fertilizer (urea, ammonium sulfate et al) and so on [18]. In the current research, we clearly observed NIs significantly increased yield and N uptake of maize compare with the application of ammonium sulfate among the three soil types, especially in DMPP and N-GD in brown soil, DMPP+N-GD in cinnamon soil and DMPP in red soil. Cui et al also found that DCD with urea can significant increased the yield of radish and N concentrations 10.4%-36.2% in vegetables [3]. A field experiment also showed that the application of DMPP increased 4.2%–4.7% and 6.6%–7.5% of rice and rape grain yield, respectively [39], which was in line with our study. NIs can maintain higher NH_4^+-N content for a long period, which improve crop more available N absorption [40]. Ammonium ions usually adsorb by clay particles or soil organic matter, thus decreasing N loss [41]. Moreover, NIs with N fertilizer can increase the synchrony between N supplied and crop demand [42]. However, the results of Huérfano et al and Guardia et al showed that there are no any effect of NIs on maize yield [43-44], which was in contrast to our results. The possible explanation is the effect of NIs mainly due to environmental factors, soil properties and the type of fertilizer [45]. The higher values of plant N uptake imply higher NUE for NIs. Obviously, NIs also significantly

improved NUE in this study. The results are agreement with He et al [46], which also found NIs with urea increased NUE.

In terms of cost, the price of DMPP is so expensive that its wide application is limited [41]. In the present study, we found that DMPP had the similar efficiency with N-GD in the brown soil, while in the cinnamon soil DMPP+N-GD was more efficient than DMPP alone, thus our best choice is the application of N-GD and DMPP+N-GD in brown soil and cinnamon soil, respectively.

Effects of soil properties on NIs use

In our study, the efficiency of NIs differed in different agricultural soils, mainly due to the different pH (brown soil: 5.3; cinnamon soil: 7.9; red soil: 4.6) and SOM. Soil pH has been considered as one of the most important factors affecting the availability of NIs, because pH has potential to effect the mobility and degradation rate of the NIs in soils [47]. In our study, the efficiency of DMPP was more stable in lower pH soil than in alkaline soil. DMPP also performs better in neutral than in alkaline soil [48], which was similar to our results. Additionally, the apparent nitrification rate in lower pH soils was lower than in alkaline soils, which is in accordance with the result of Lu et al [31]. In addition to soil pH, the effectiveness of NIs also influenced by SOM. SOM can absorb NIs and provide energy source for the microorganisms, which leads to the degradation of NIs, decreases the ability of NIs to inhibit nitrification [49]. The results was similarly to our study, SOM was higher in cinnamon soil than both brown and red soil, thus the efficiency of DMPP in cinnamon soil was lower than the other two soils. But the effect of DMPP+N-GD had little influenced by SOM.

In addition, there is less study of N-GD, so more research should be required to understand its effect and mechanism. Moreover, the effect of NIs on the N cycle in a range of soil types, cultivated

vegetation types, and climatic condition should be fully elucidated.

Conclusions

In our study, we clearly observed NIs with ammonium sulfate can significantly influence soil inorganic nitrogen, improve yield and NUE. DMPP and N-GD had the same efficiency of decreasing soil nitrification, improving yield and NUE of maize in the brown soil, while NIs combination DMPP+N-GD was the best way to increase soil available nitrogen and improving NUE in the cinnamon soils, DMPP was the most efficiency in lowering soil nitrification and increasing maize yield and NUE in the red soil. The study shows that the effect of different NIs varied among different soils, and soil pH is the main factor.

Acknowledgements

We are grateful to the National Field Research Station of Shenyang Agroecosystems, Chinese Academy of Sciences, for providing the experimental field.

References

1. Sutton MA, Howard CM, Erisman JW. The European Nitrogen Assessment: Sources, Effects and Policy Perspectives. Cambridge University Press; 2011.

2. Coskun D, Britto DT, Shi W and Kronzucker HJ. Nitrogen transformations in modern agriculture and the role of biological nitrification inhibition. Nat. Plants. 2017; 3: 17074 doi:10.1038/nplants.2017.74.

3. Cui M, Sun XC, Hu CX, Hong J, Di HJ, Tan QL, et al. Effective mitigation of nitrate leaching and nitrous oxide emissions in intensive vegetable production systems using a nitrification inhibitor, dicyandiamide. J. Soil Sediment. 2011; 11: 722–730.

4. Moir JL, Malcolm BJ, Cameron KC, Di HJ. The effect of dicyandiamide on pasture nitrate

concentration, yield and N offtake under high N loading in winter and spring. Grass Forage Sci. 2012; 67: 391–402.

5. Haynes RJ, Goh KM. Ammonium and nitrate nutrition of plants. Biol. Rev. 1978; 53: 465–510.

6. Hu HW, Chen D, He JZ. Microbial regulation of terrestrial nitrous oxide formation: understanding the biological pathways for prediction of emission rates. FEMS Microbiol. Rev. 2015; 39: 729–749.

7. Weiske A, Benckiser G, Herbert T, Ottow J. Influence of the nitrification inhibitor 3, 4-dimethylpyrazole phosphate (DMPP) in comparison to dicyandiamide (DCD) on nitrous oxide emissions, carbon dioxide fluxes and methane oxidation during 3 years of repeated application in field experiments. Biol. Fert. Soils. 2001; 34: 109–117.

8. Majumdar D, Pathak H, Kumar S, Jain MC. Nitrous oxide emission from a sandy loam Inceptisol under irrigated wheat in India as influenced by different nitrification inhibitors. Agr. Ecosyst. Environ. 2002; 91: 283–293.

9. Zaman M, Saggar S, Blennerhassett JD, Singh J. Effect of urease and nitrification inhibitors on N transformation, gaseous emissions of ammonia and nitrous oxide, pasture yield and N uptake in grazed pasture system. Soil Biol. Biochem. 2009; 41: 1270–1280.

10 Di HJ, Cameron KC. Inhibition of ammonium oxidation by a liquid formulation of 3, 4-Dimethylpyrazole phosphate (DMPP) compared with a dicyandiamide (DCD) solution in six New Zealand grazed grassland soils. J. Soil Sediment. 2011; 11: 1032–1039.

11. Pfab H, Palmer I, Buegger F, Fiedler S, Muller T, Ruser R. Influence of a nitrification inhibitor and of placed N fertilization on N_2O fluxes from a vegetable cropped loamy soil. Agr. Ecosyst. Environ. 2012; 150: 91–101.

12. Shi X, Hu HW, Muller C, He JZ, Chen D, Suter HC. Effects of the nitrification inhibitor 3, 4-dimethylpyrazole phosphate on nitrification and nitrifiers in two contrasting agricultural soils. Appl. Environ. Microb. 2016; 82: 5236–5248.

13. McCarty GW. Modes of action of nitrification inhibitors. Biol. Fert. Soils. 1999; 29: 1–9.

14. Gao JC, Luo JF, Lindsey S, Shi YL, Sun ZL, Wei ZB, et al. Benefits and risks for the environment and crop production with application of nitrification inhibitors in China. J. Soil Sci. Plant Nut. 2021; 21: 497–512.

15. Giltrap DL, Singh J, Saggar S, Zaman M. A preliminary study to model the effects of a nitrification inhibitor on nitrous oxide emissions from urine-amended pasture. Agr. Ecosyst. Environ. 2010; 136: 310–317.

16. Zerulla W, Barth T, Dressel J, Erhardt K, Horchler von Locquenghien K, Pasda G, et al. 3, 4-dimethylpyrazole phosphate (DMPP)–a new nitrification inhibitor for agriculture and horticulture. Biol. Fert. Soils. 2001; 34: 79–84.

17. Mahmood T, Ali R, Latif Z, Ishaque W.Dicyandiamide increases the fertilizer N loss from an alkaline calcareous soil treated with ^{15}N-labelled urea under warm climate and under different crops. Biol. Fert. Soils. 2011; 47: 619–631.

18. Guardia G, Marsden KA, Vallejo A, Jones DL, Chadwick DR. Determining the influence of environmental and edaphic factors on the fate of the nitrification inhibitors DCD and DMPP in soil. Sci. Total Environ. 2018; 624: 1202–1212.

19. Subbarao GV, Ito O, Sahrawat KL, Berry WL, Nakahara K, Ishikawa T, et al. Scope and strategies for regulation of nitrification in agricultural systems—challenges and opportunities. Crit. Rev. Plant Sci. 2006; 25: 303–335.

20. Fan C, Li B, Xiong Z. Nitrification inhibitors mitigated reactive gaseous nitrogen intensity in intensive vegetable soils from China. Sci. Total Environ. 2018; 612: 480–489.

21. Dong XX, Zhang LL, Wu ZJ, Zhang HW, Gong P. The response of nitrifier, N-fixer and denitrifier gene copy numbers to the nitrification inhibitor 3,4-dimethylpyrazole phosphate. Plant Soil Environ. 2013; 59: 398–403.

22. Dong D, Kou Y, Yang W, Chen G, Xu H. Effects of urease and nitrification inhibitors on nitrous oxide emissions and nitrifying/denitrifying microbial communities in a rainfed maize soil: a 6-year field observation. Soil Till. Res. 2018; 180: 82–90.

23. Zhao Z, Wu D, Bol R, Shi Y, Guo Y, Meng F, et al. Nitrification inhibitor's effect on mitigating N_2O emissions was weakened by urease inhibitor in calcareous soils. Atmos. Environ. 2017; 166: 142–150.

24. Pasda G, Hähndel R, Zerulla W. Effect of fertilizers with the new nitrification inhibitor DMPP (3,4-dimethylpyrazole phosphate) on yield and quality of agricultural and horticultural crops. Biol. Fert. Soils. 2001; 34: 85–97.

25. Ahmed M, Yu WJ, Lei M, Raza S, Zhou JB. Mitigation of ammonia volatilization with application of urease and nitrification inhibitors from summer maize at the Loess Plateau. Plant Soil Environ. 2018; 64: 164–172.

26. Tao R, Li J, Guan Y, Liang Y, Hu B, Lv J, et al. Effects of urease and nitrification inhibitors on the soil mineral nitrogen dynamics and nitrous oxide (N_2O) emissions on calcareous soil. Environ. Sci. Pollut. R. 2018; 25: 9155–9164.

27. Yang LJ, Zhang LL, Geisseler D, Wu ZJ, Gong P, Xue Y, et al. Available C and N affect the utilization of glycine by soil microorganisms. Geoderma 2016; 283: 32–38.

28. Zhao ZQ, Zhu YG, Li HY, Smith SE, Smith FA. Effects of forms and rates of potassium fertilizers on cadmium uptake by two cultivars of spring wheat (*Triticum aestivum*, L.). Environ. Int. 2004; 29: 973–978.

29. Kempers AJ, Luft AG. Re-examination of the determination of environmental nitrate as nitrite by reduction with hydrazine. Analyst. 1988; 113: 1117–1120.

30. Moll RH, Kamprath EJ, Jackson WA. Analysis and interpretation of factors which contribute to efficiency of nitrogen utilization. Agron. J. 1982; 74: 562–564.

31. Lu Y, Zhang X, Jiang J, Kronzucker HJ, Shen W, Shi W. Effects of the biological nitrification inhibitor 1,9-decanediol on nitrification and ammonia oxidizers in three agricultural soils. Soil Biol. Biochem. 2019; 129: 48–59.

32. Briggs, GG. The behavior of the nitrification inhibitor "N-Serve" in broadcast and incorporated applications to soil. J. Sci. Food Agric. 1975; 26: 1083–1092.

33. Guo YJ, Di HJ, Cameron KC, Li B. Effect of application rate of a nitrification inhibitor, dicyandiamide (DCD), on nitrification rate, and ammonia-oxidizing bacteria and archaea growth in a grazed pasture soil: an incubation study. J. Soil Sediment. 2014; 14: 897–903.

34. Gong P, Zhang LL, Wu ZJ, Chen ZH, Chen LJ, Responses of ammonia-oxidizing bacteria and archaea in two agricultural soils to nitrification inhibitors DCD and DMPP: a pot experiment. Pedosphere 2013; 23: 729–739.

35. Benckiser G, Christ E, Herbert T, Weiske A, Blome J, Hardt M. The nitrification inhibitor 3, 4-dimethylpyrazole-phosphat (DMPP)-quantification and effects on soil metabolism. Plant Soil 2013; 371: 257–266.

36. Fangueiro D, Fernandes A, Coutinho J, Moreira N, Trindade H. Influence of two nitrification

inhibitors (DCD and DMPP) on annual ryegrass yield and soil mineral N dynamics after incorporation with cattle slurry. Commun. Soil Sci. Plan. 2009; 40: 3387–3398.

37. Zaman M, Di HJ, Cameron KC. Gross N-mineralization and nitrification rates and their relationships to enzyme activities and soil microbial biomass in soils treated with dairy shed effluent and ammonium fertilizer in the field. Soil Use Manage. 1999; 15: 188–194.

38. Ma Q, Wu ZJ, Shen SM, Zhou H, Jiang CM, Xu YG, et al. Responses of biotic and abiotic effects on conservation and supply of fertilizer N to inhibitors and glucose inputs. Soil Biol. Biochem. 2015; 89: 72–81.

39. Li H, Liang XQ, Chen YX, Lian YF, Tian GM, Ni WZ. Effect of nitrification inhibitor DMPP on nitrogen leaching, nitrifying organisms, and enzyme activities in a rice-oilseed rape cropping system. J. Environ. Sci. 2008; 20: 149–155.

40. Patra AK, Chhonkar PK, Khan MA. Effect of green manure Sesbania sesban and nitrification inhibitor encapsulated calcium carbide (ECC) on soil mineral-N, enzyme activity and nitrifying organisms in a rice-wheat cropping system. Eur. J. Soil Biol. 2006; 42: 173–180.

41. Yang M, Fang Y, Sun D, Shi Y. Efficiency of two nitrification inhibitors (dicyandiamide and 3, 4-dimethypyrazole phosphate) on soil nitrogen transformations and plant productivity: a meta-analysis. Sci. Rep. 2016; 6(1): doi:10.1038/srep22075.

42. Ma Q, Wu ZJ, Yu WT. Does the incorporation of dicyandiamide and hydroquinone with straw enhance the nitrogen supplying capacity in soil? Appl. Soil Ecol. 2019; 136: 158–162.

43. Huérfano X, Estavillo JM, Fuertes-Mendizábal T, Torralbo F, González-Murua C, Menendez S. DMPSA and DMPP equally reduce N_2O emissions from a maize-ryegrass forage rotation under Atlantic climate conditions. Atmos. Environ. 2018; 187: 155–165.

44. Guardia G, Cangani MT, Andreu G, Sanz-Cobena A, García-Marco S, Álvarez JM, et al. Effect of inhibitors and fertigation strategies on GHG emissions, NO fluxes and yield in irrigated maize. Field Crop Res. 2017; 204: 135–145.

45. Barth G, Von Tucher S, Schmidhalter U. Effectiveness of 3, 4-Dimethylpyrazole Phosphate as nitriflcation inhibitor in soil as influenced by inhibitor concentration, application form, and soil matric potential. Pedosphere. 2008; 18: 378–385.

46. He T, Liu D, Yuan J, Luo J, Lindsey S, Bolan N, et al. Effects of application of inhibitors and biochar to fertilizer on gaseous nitrogen emissions from an intensively managed wheat field. Sci. Total Environ. 2018; 628-629: 121–130.

47. Zhang HJ, Wu ZJ, Zhou QX. Dicyandiamide sorption-desorption behaviour on soils and peat humus. Pedosphere. 2004; 14: 395–399.

48. Liu R, Hayden H, Suter H, He JZ and Chen DL, The effect of nitrification inhibitors in reducing nitrification and the ammonia oxidizer population in three contrasting soils. J. Soil Sediment. 2015; 15: 1113–1118.

49. Goring CAI, Control of nitrification by 2-chloro-6-(trichloromethyl) pyridine. Soil Sci. 1962; 93: 211–218.

Figure

Click here to access/download;Figure;Fig1.tif

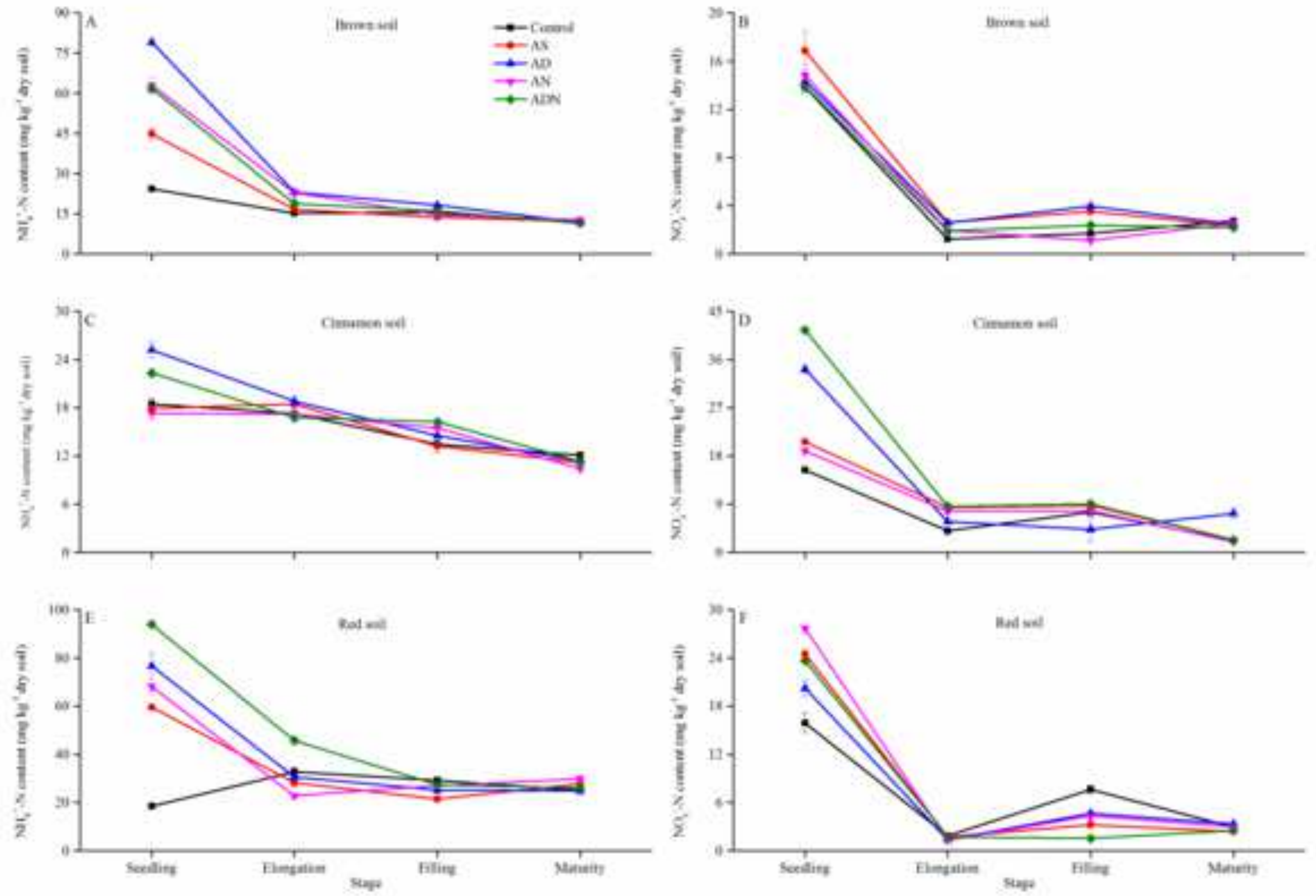

Figure

Click here to access/download;Figure;Fig2.tif

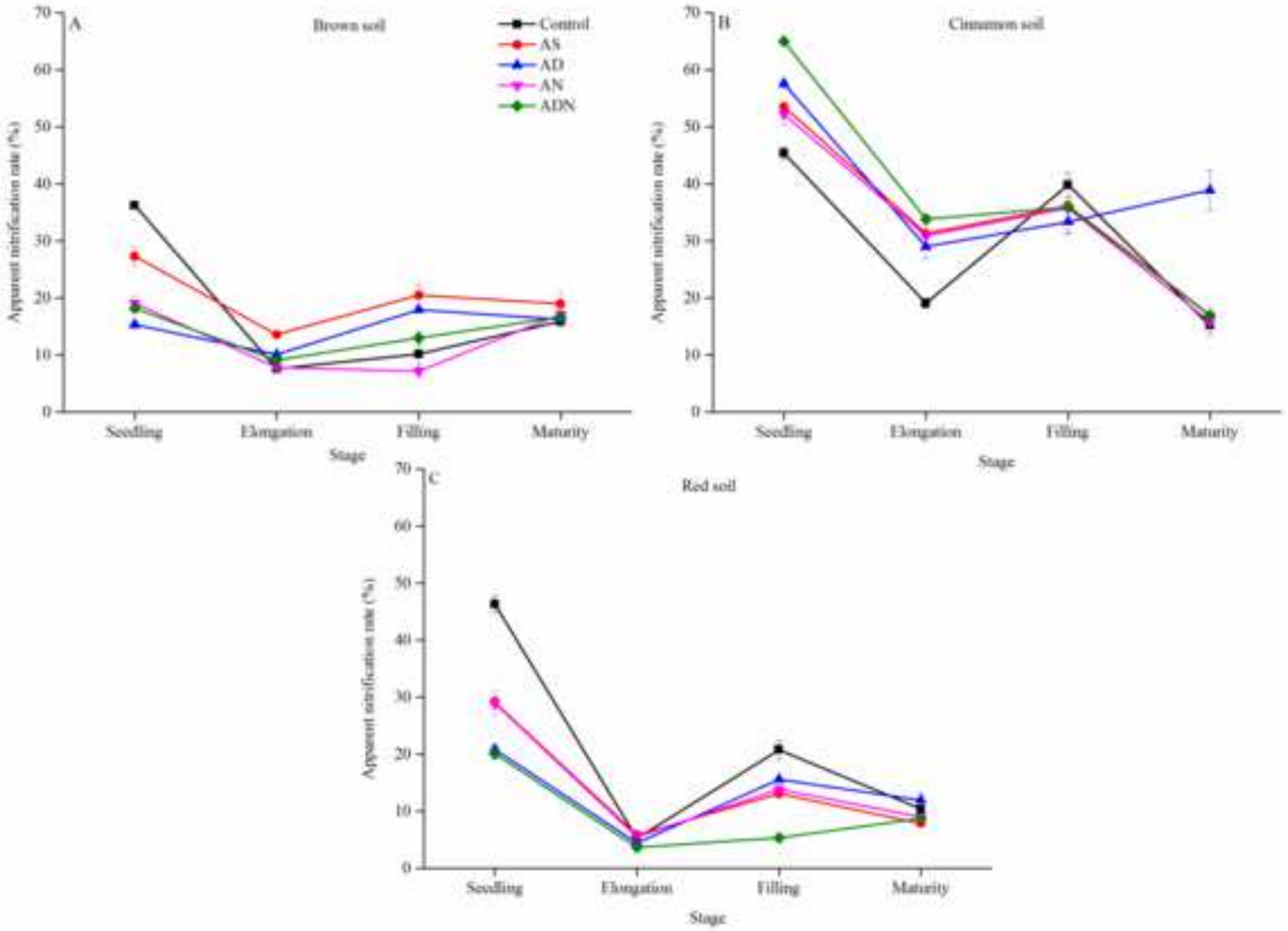

Figure

Click here to access/download;Figure;Fig3.tif

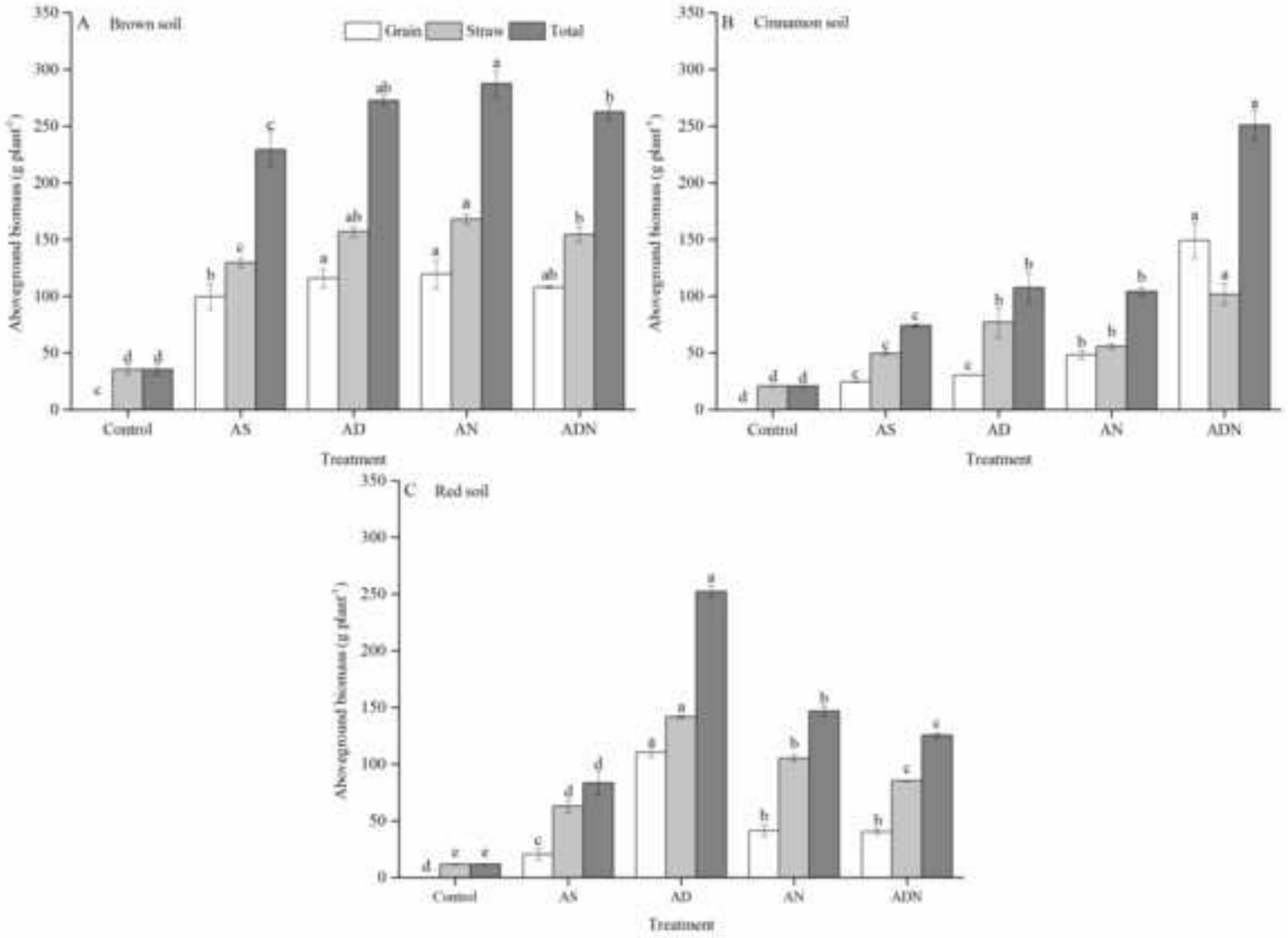

Figure

Click here to access/download;Figure;Fig4.tif

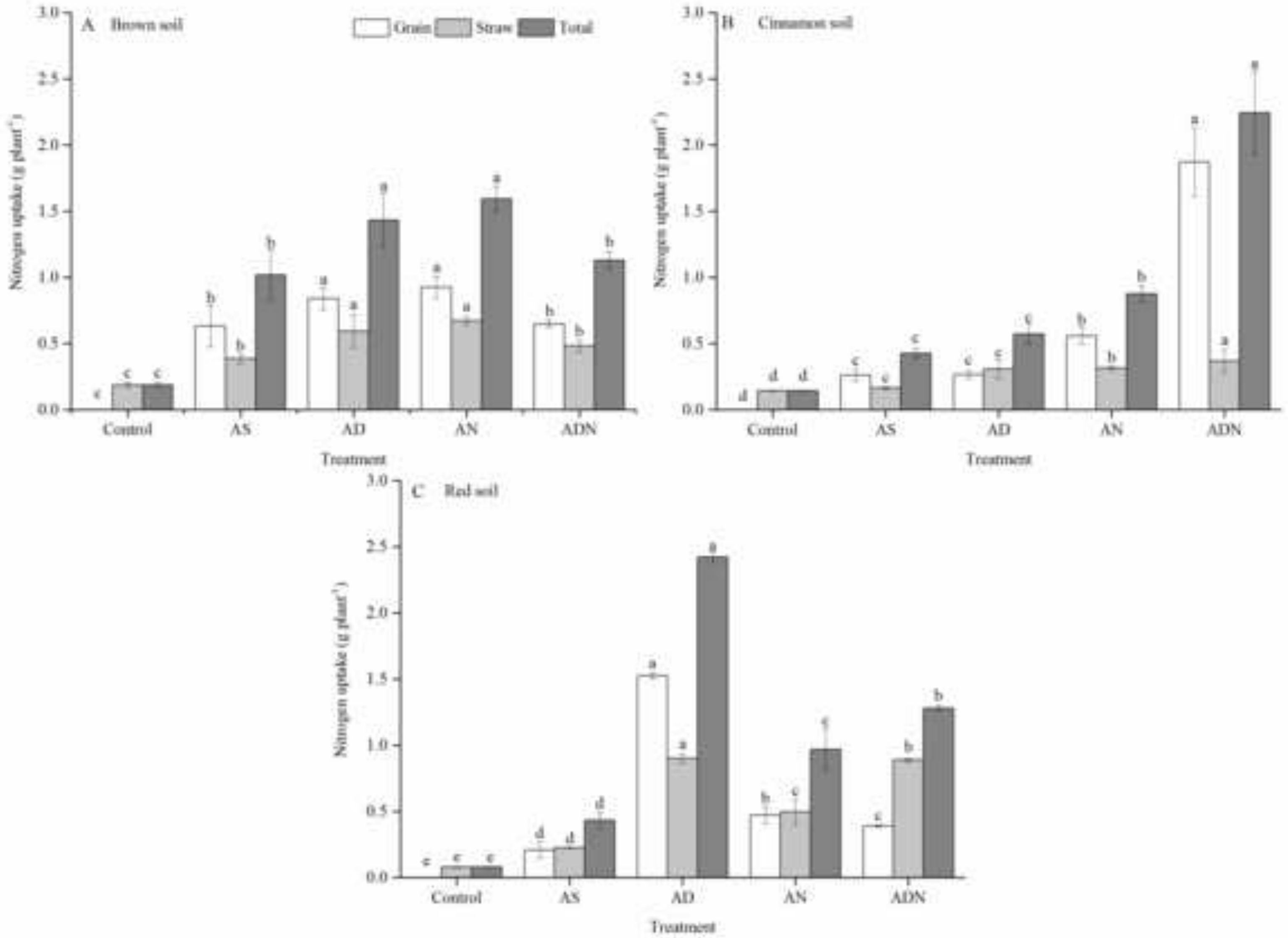

Figure

Click here to access/download;Figure;Fig5.tif

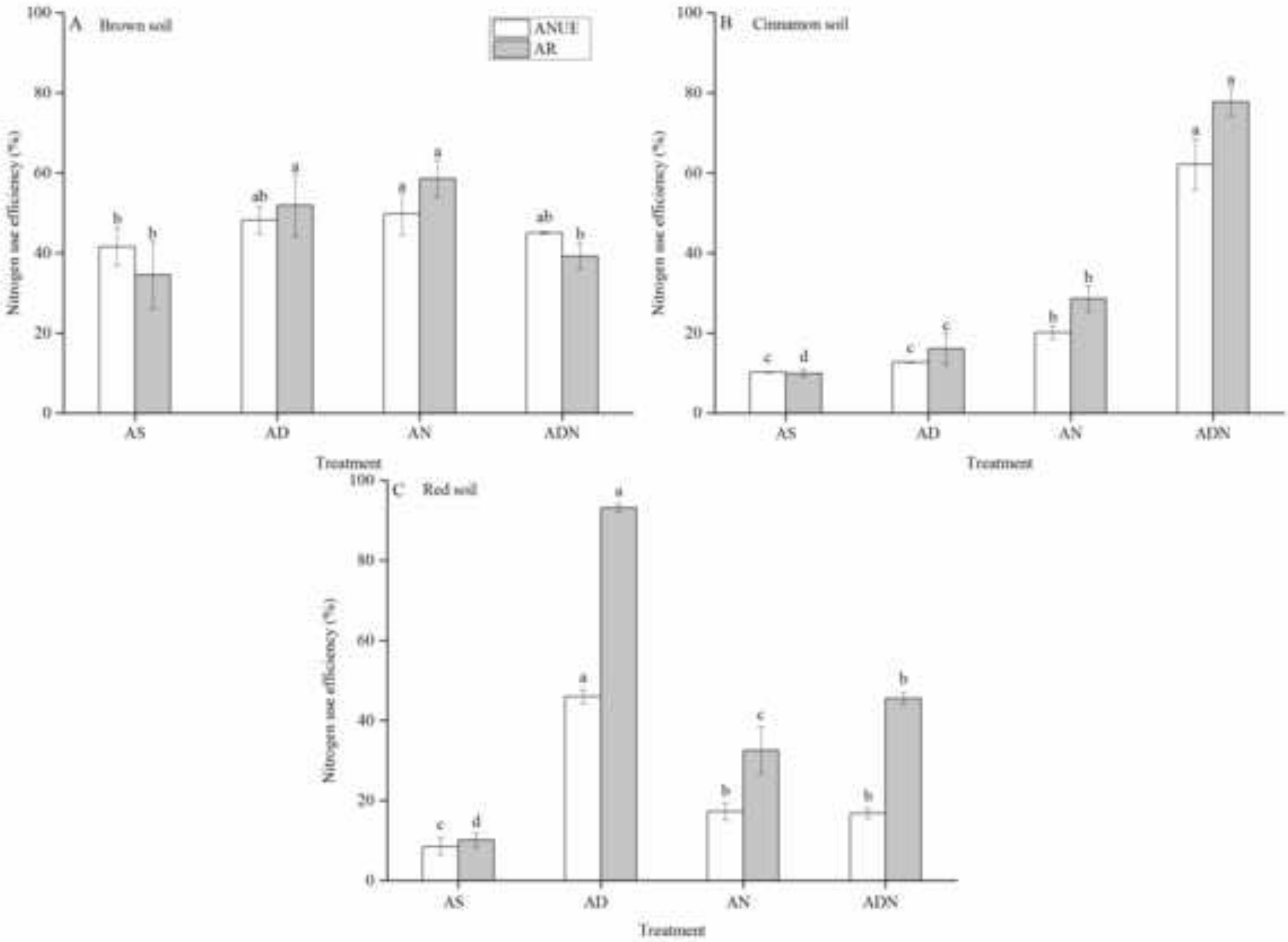

Supplement: Attachment — Submitted filename: PONE-D-21-09541_R3-reviewer-manuscript.pdf [file pone.0272935.s007.pdf]
