# Supplementary material for: Effects of combined nitrification inhibitors on soil nitrification, maize yield and nitrogen use efficiency in three agricultural soils
Source: PLoS One. 2022 Aug 22;17(8):e0272935. doi: 10.1371/journal.pone.0272935 (PMC9394818; doi:10.1371/journal.pone.0272935)
Supplement: Attachment — Submitted filename: Review comments for.docx [file pone.0272935.s002.docx]

**Review comments for** PONE-D-21-09541

**“Effects of combined nitrification inhibitors on soil nitrification, maize yield and nitrogen use efficiency in three agricultural soils”**

To understand the efficiency of nitrification inhibitors with N fertilizer on soil nitrification, yield and nitrogen use efficiency of maize (*Zea mays* L.), an outdoor pot experiment with different nitrification inhibitors in three soils with different pH were conducted in the paper. The reviewer think that the problems of this paper are as follows:

1.There are some sentences with irregular grammar or incomplete meaning: Such as:

(1)L39:”DMPP and N-GD were more effective in the red soil than in the cinnamon soil”What is more effective?

(2)L40 and 41:“… than other two soils, … than other two soils” were changed to“…than that in other two soils,… than that in other two soils.”

(3)L169:“NH4+-N content and NO3--N content” should be changed to“Contents of NH4+-N and NO3—N in soils”.

(4)L197:“Apparent nitrification rate (ANR) indicates that intensify of soil nitrification”, the sentence is not a clause, it should be changed to “Apparent nitrification rate (ANR) indicates the intensify of soil nitrification” .

(5)L253:“Agronomy nitrogen use efficiency and apparent nitrogen recovery” should be changed to“Utilization efficiency of agronomy ang recovery of apparent nitrogen”.

(6) L327:“was more stable in lower pH soil than in alkaline soil” should be changed to“was more stable in lower pH soil than that in alkaline soil”.

2.Improper expression or incomplete meaning of some section titles:

(1)L146:“Analytical procedures” should be changed to “Analytical methods” (Only the determination methods are given, and there are no determination procedures or operation processes)

(2)Note: To distinguish the relationship between the instrument and the method, the instrument only is the means of the method, but the instrument itself is not the method. Such as: “with a pH meter” in L147, “with an elemental analyzer” in L148, “determined by the flame photometer method” in L150-151, and “using a Continuous Flow Analyzer” in L151-152. Above all, these sentences should be expressed in the form of “methods” rather than “instruments”, especially, “determined by the flame photometer method” in L150-151 was a wrong expression, should be changed to “determined by the flame photometry method”.

(3) The meaning of “Apparent nitrification rate”in L196 was too general.

3.Some tense usageswere not correct,the past tense should be used in many sentences but used the present tense.

4.In the description of result and content discussion, the first person is used too much. Such as: "we conduct an outdoor.." in L99, “We clearly observed that…..” in L170(Figure 1 should be described first), “We also found that…” in L179, “we clearly observed……” in L340(It is more appropriate to have such a statement in the conclusion). It is suggested that the result or content described in the first person should be described in the passive voice.

5.“In our study” is used too often, which is to discuss the results of this paper, so it is unnecessary to emphasize it many times.

6. The expression of this comparison "The results was similarly to our study,....."in L332 is improper, it should be expressed "The results of this paper was similarly to that of the literature,.....".

7.The part of “conclusion” is an objective statement of the law presented by the research results, The expression of “In our study, we clearly observed” should be deleted.

8.The logical relationship between several experimental results in the result part is not very clear, moreover, the logical relationship between the results and the discussion is also fuzzy.
