# Supplementary material for: Effects of combined nitrification inhibitors on soil nitrification, maize yield and nitrogen use efficiency in three agricultural soils
Source: PLoS One. 2022 Aug 22;17(8):e0272935. doi: 10.1371/journal.pone.0272935 (PMC9394818; doi:10.1371/journal.pone.0272935)
Supplement: Attachment — Submitted filename: Response to reviewers.docx [file pone.0272935.s003.docx]

Dear Manuel Joaquín Reigosa, Ph.D.,

Thank you very much for your letter and the reviewers’ reports concerning our manuscript entitled “PONE-D-21-09541”. Those comments are valuable and very helpful for revising and improving our paper. We have read through comments carefully and have made corrections. Based on the instructions provided in your letter, we uploaded the file of the revised manuscript. Revisions in the text are shown using red highlight for easy checking purpose. The responses to the reviewer's comments are presented following.

**Response to academic editor’s comments**

1. Please ensure that your manuscript meets PLOS ONE's style requirements, including those for file naming.

We ensured that our manuscript meets PLOS ONE's style requirements, including those for file naming.

2. Thank you for stating the following in the Competing Interests section:

"The authors have declared that no competing interests exist."

We note that one or more of the authors are employed by a commercial company: North Huajin Chemical Industries Group Corporation, Jinxi Natural Gas Chemical Co. Ltd.

We ensured that we have specifically and accurately indicated the role(s) that these authors had in our study. We confirmed that this commercial affiliation does not alter your adherence to all PLOS ONE policies on sharing data and materials by including the following statement.

3. PLOS requires an ORCID iD for the corresponding author in Editorial Manager on papers submitted after December 6th, 2016. Please ensure that you have an ORCID iD and that it is validated in Editorial Manager.

We ensured that we have an ORCID iD and that it is validated in Editorial Manager.

**Response to reviewers’ comments**

**Reviewer #1:** This manuscript described the effects of different nitrification inhibitors on soil nitrification and maize yield in various agricultural soils with different physicochemical properties. The subject is interesting and the paper is well organized. The introduction gave a satisfactory literature survey. The research methods used in this paper were described in detail and the data was well statistically analyzed. Appropriate figures were given to make the paper understood easily.
From my point of view, the work is well-done and provides theoretical basis to utilize fertilizer nitrogen effectively on maize in three soils with different nitrification inhibitors. The paper conclusions provided critical support for the theory that different nitrification inhibitors have different effects in different physical and chemical properties of soils. In addition, it seeks to find an economical way to improve NUE with new nitrification inhibitor N-GD due to higher price of DMPP. The results also showed that soil pH plays an important role in the effectiveness of DMPP, N-GD and their combination in influencing soil nitrification, yield and NUE of maize. Therefore, this article deserves to be published.
Some minor modifications should be made before publication:
RESPONSE: We appreciate the suggestions from the reviewer, which help us greatly improve our manuscript.

1. In this manuscript, "decrease nitrification" should be revised as “inhibit/suppress nitrification".

It was revised.
2. Page 2, line 24, please revise “The application” to “ Application”.

It was revised.
3. Page 2, line 25, please revise “the most efficiency” to “the most efficient”.

It was revised.
4. Page 2, line 38, please add “that” after “than”.

It was added.
5. Page 3, line 59, please delete “which”.

It was deleted.
6. Page 4, line 78, please add “and” in front of “field”.

It was added.
7. Page 4, line 82, please add parentheses.

It was added.
8. Page 5, line 90, please delete “are”.

It was deleted.
9. Page 7, line 139, please revise “sampling” to “samples”.

It was revised.
10. Page 11, line 216, Page 16, line 332, please revise “was” to “were”.

It was revised.
11. Page 17, line 341, please add “of maize” after “NUE”.

It was added.
12. Page 17, line 343, “improving” should be revised to “improve”.

It was revised.

**Reviewer #2:** To understand the efficiency of nitrification inhibitors with N fertilizer on soil nitrification, yield and nitrogen use efficiency of maize (*Zea mays* L.), an outdoor pot experiment with different nitrification inhibitors in three soils with different pH were conducted in the paper. The reviewer think that the problems of this paper are as follows:

RESPONSE: We appreciate the suggestions from the reviewer, which help us greatly improve our manuscript.
1. There are some sentences with irregular grammar or incomplete meaning: Such as:
(1) L39:”DMPP and N-GD were more effective in the red soil than in the cinnamon soil” What is more effective?

After careful checking, it was changed “DMPP was more effective in the red soil than in the cinnamon soil”.
(2)L40 and 41:“… than other two soils, … than other two soils” were changed to“…than that in other two soils,… than that in other two soils.”

It was changed.
(3)L169:“NH_4_^+^-N content and NO_3_^-^-N content” should be changed to“Contents of NH_4_^+^-N and NO_3_^-^-N in soils”.

It was changed.
(4)L197:“Apparent nitrification rate (ANR) indicates that intensify of soil nitrification”, the sentence is not a clause, it should be changed to “Apparent nitrification rate (ANR) indicates the intensify of soil nitrification”.

It was changed.
(5)L253:“Agronomy nitrogen use efficiency and apparent nitrogen recovery” should be changed to“Utilization efficiency of agronomy ang recovery of apparent nitrogen”.

It was changed.
(6)L327:“was more stable in lower pH soil than in alkaline soil” should be changed to“was more stable in lower pH soil than that in alkaline soil”.

It was changed.
2. Improper expression or incomplete meaning of some section titles:
(1)L146:“Analytical procedures” should be changed to “Analytical methods” (Only the determination methods are given, and there are no determination procedures or operation processes)

It was changed.
(2)Note: To distinguish the relationship between the instrument and the method, the instrument only is the means of the method, but the instrument itself is not the method. Such as: “with a pH meter” in L147, “with an elemental analyzer” in L148, “determined by the flame photometer method” in L150-151, and “using a Continuous Flow Analyzer” in L151-152. Above all, these sentences should be expressed in the form of “methods” rather than “instruments”, especially, “determined by the flame photometer method” in L150-151 was a wrong expression, should be changed to “determined by the flame photometry method”.

We have rewrite these sentences to express in the form of “methods”. It was changed.
(3) The meaning of “Apparent nitrification rate” in L196 was too general.

We revised the meaning of “Apparent nitrification rate” to make it more specific.
3. Some tense usages were not correct, the past tense should be used in many sentences but used the present tense.

It was corrected.
4. In the description of result and content discussion, the first person is used too much. Such as: "we conduct an outdoor.." in L99, “We clearly observed that…..” in L170(Figure 1 should be described first), “We also found that…” in L179, “we clearly observed……” in L340(It is more appropriate to have such a statement in the conclusion). It is suggested that the result or content described in the first person should be described in the passive voice.

We fully agreed with the suggestion of the reviewer. According the suggestions, we described in the passive voice to describe the result or content described in the first person. Such as “an outdoor… was conducted” . The first person have been deleted. In the conclusion, we deleted the sentence “we clearly observed……”.
5. “In our study” is used too often, which is to discuss the results of this paper, so it is unnecessary to emphasize it many times.

We fully agree with the suggestion of the reviewer. According the suggestions, we deleted “In our study”.
6. The expression of this comparison "The results was similarly to our study,....."in L332 is improper, it should be expressed "The results of this paper was similarly to that of the literature,.....".

It was changed.
7. The part of “conclusion” is an objective statement of the law presented by the research results, The expression of “In our study, we clearly observed” should be deleted.

We deleted the expression of “In our study, we clearly observed”.
8. The logical relationship between several experimental results in the result part is not very clear, moreover, the logical relationship between the results and the discussion is also fuzzy.

We reorganized the results and the discussion to make the content clear.

**Reviewer #3:** Opinion on manuscript: PONE-D-21-09541 major revision
Global food security is a key challenge of the modern world. The demand for food is growing exponentially and in this regard agricultural production has to be increased to meet global food demands. One of the key agricultural practices is the higher application of nitrogenous fertilizers. However, the use efficiency of applied fertilizers is currently very low. This low efficiency is attributed to the great losses of nitrogenous fertilizers after their application into the soil due to leaching, run off, volatilization and de-nitrification. Overall, the findings of this study have essential implications for the enhancing NUE and apparent nitrogen recovery. The comments are as follows, and most of them are suggestions to the authors that should be taken on board for a revised version.

RESPONSE: We appreciate the suggestions from the reviewer, which help us greatly improve our manuscript.
Specific comments:
1. Page 2 - line 25 –“efficiency ways” should be changed into “efficient ways”
It was changed.
2. Page 2 - line 32 –“which decreased apparent nitrification rate 28%” should be changed into “which decreased apparent nitrification rate by 28%”
It was changed.
3. Page 2 - line 35 –“5.07 times” delete “times”
It was deleted.
4. Page 4 - line 67,69, 88 – “But” “And” should not be the first word of the sentence and rewrite this.
We have already rewritten this.
5. Page 4 - line 81 – “A laboratory incubation experiment…” should be marked with a reference.
We have already marked the reference.
6. Page 8 - line 147 – “pH meter” should be marked with the mode.
We have already marked the mode.
7. Page 8 – line 149 – “molybdenum blue method” reference?
The reference has been marked.
8. Page 10 - line 197 – “Apparent nitrification rate” should be abbreviated.
We abbreviated “Apparent nitrification rate” as “ANR”.
9. Page 13 - line 255 – “Agronomy nitrogen use efficiency” should be changed into “agronomic nitrogen use efficiency”.

It was changed “Agronomic nitrogen use efficiency”.
10. Page 14 - line 289 – “which was line in with” Grammatical errors
It was corrected.
10. Page 15 - line 295-297 the discussion is so simple, rewrite this part.
We have rewritten this part.
10. Page 14 - line 314 – “environmental factors, soil properties and the type of fertilizer” should be more detailed. Rewrite this part according to the results of this article.
We have rewritten this part according to the results of this article.
10. Page 16- line 323 – “In our study, the efficiency of NIs differed in different agricultural soils, mainly due to the different pH”. The conclusion is far fetched and rewrite this sentence.
The effects of pH on nitrification should be more detailed.
We have rewritten this sentence. The effect of pH on nitrification has been described in detail.
11. Page 16- line 322 – Please add a “Table for correlation analysis between soil properties and NIs use” if possible.

The application of NIs have the potential to slow soil nitrification, thus affecting soil inorganic (NH_4_^+^-N and NO_3_^-^-N) concentrations. Therefore, we added a “Table for correlation analysis among soil properties, NH_4_^+^-N and NO_3_^-^-N”.
12. please calculate the nitrification inhibition rate of NIs
We already calculated the nitrification inhibition rate of NIs, and added a table in the supplementary material.
13. The whole manuscript some of the expressions are too colloquial and the meaning is not clearly expressed, please check the grammar in the article carefully.

We revised some of the expression of the manuscript to make the content clear. We checked the grammar in the article carefully, and corrected it.

Reviewer #4: This manuscript had compared the effects of combined nitrification inhibitors on soil nitrification, maize yield and nitrogen use efficiency in three agricultural soils with pot experiment. Compared to the other studies, this research is not novel. Other issues that need revision or clarification include:

RESPONSE: We appreciate the suggestions from the reviewer, which help us greatly improve our manuscript. The paper aims to compare the different effects of nitrification inhibitors in different physical and chemical properties of soils, and this is the first research to study the effect of new nitrification inhibitor (nitrogen protectant (N-GD)) in different soils.
(1) In introduction: It mentioned in line 67-68 that "there has been little research on the effect of NIs combined with ammonium sulfate in soils". However, in this study, they did not compare ammonium sulfate with other N fertilizers, such as urea, or NH4Cl. It's better to summarize other studies in lines 82-90.

We summarized other studies in the revised manuscript.
(2)M&M section：I suggest to arrange the orders of the three soils as Cinnamon soil, Brown soil, Red soil, i.e based on soil pH, from high to low, because soil pH is an very important parameter to affect nitrification.
The available K of Brown soil in Table 1 was as low as 5.7 mg/kg. It is too low. Please check it.

We fully agreed with the suggestion of the reviewer. We arranged the orders of the three soils as Cinnamon soil, Brown soil, Red soil. We checked the available K of brown soil, and corrected it.
(3) Result: the pixels of the Figures are too low!

We adjusted the pixels of the Figures.
(4) Discussion: The mechanism behind the results need analysis.
Line 281-282,"It is worth to mention that nitrification in red soil becomes stronger, which was according with Lu et al [31], mainly due to the tillage during the decades." It seems that the nitrification of red soil was lower than the two other soils.

It is well known that nitrification in natural red soil is extremely weak due to low pH and soil texture, but nitrification has been accelerated in many such soils following tillage in recent decades. However, the nitrification of red soil was lower than the two other soils. The reason of this phenomenon is its lower pH and soil organic matter.

We would like to thank you for allowing us to submit a revised manuscript with major revision and we highly appreciate your time and consideration.

Sincerely,

Professor Dongpo Li
