# Supplementary material for: Effects of combined nitrification inhibitors on soil nitrification, maize yield and nitrogen use efficiency in three agricultural soils
Source: PLoS One. 2022 Aug 22;17(8):e0272935. doi: 10.1371/journal.pone.0272935 (PMC9394818; doi:10.1371/journal.pone.0272935)
Supplement: Attachment — Submitted filename: Review comments for D 21-09541-20210825.docx [file pone.0272935.s004.docx]

**Comments on the second review for** PONE-D-21-09541

**“Effects of combined nitrification inhibitors on soil nitrification, maize yield and nitrogen use efficiency in three agricultural soils”**

**Comments on the second review:**

The following points:

1. Recommendation (Answer options: Minor Revision before accept)

2. Is the manuscript technically sound, and do the data support the conclusions? (Answer options: Yes)

3. Has the statistical analysis been performed appropriately and rigorously? (Answer options: Yes,)

4. Does the manuscript adhere to the PLOS Data Policy? Additional details can be found at http://journals.plos.org/plosone/s/materials-and-software-sharing. (Answer options: Yes)

5. Is the manuscript presented in an intelligible fashion and written in standard English? (Answer options: Yes)

6. Review Comments to the Author (minimum 100 characters)

The author is very serious about the revision of the paper and can revise the manuscript according to the suggestions or opinions of the reviewer, but he still needs to pay attention to some details of the English expression.

7. PLOS authors have the option to publish the peer review history of their article (https://journals.plos.org/plosone/s/editorial-and-peer-review-process#loc-peer-review-history). If published, this will include your full peer review and any attached files. Do you want your identity to be public for this peer review? (Answer options: No)

7a. If you answered "Yes" above, that you would like your identity to be public, please provide your full name, as it should appear on the published peer review. Please do not sign the review on behalf of another person.

Confidential to Editor:

1. Do you have any potentially competing interests? If none, type "None." Our policy on competing interests can be found at http://journals.plos.org/plosone/s/competing-interests. (Answer options: "None.")

2. Did you receive any assistance in preparing this review (e.g. from a post-doc or graduate student)? (Answer options: No)

The manuscript has been carefully revised by the author, but there are still several small problems as follows：

1. L26 and L102,103: The active voice such as “we conducted an outdoor pot ……” used in the abstract L26 is inconsistent with the passive voice such as “an outdoor pot experiment with different types of NIs additions in the above three soils was conducted” in the preface L102,103. It suggested that the passive voice should be used as much as possible in the full text.

2.L65，67: There are no percentage signs for some quantities such as “58.5 in L65” and “83.8” that need to be added with a percentage sign “%”, i.e. change 58.5 to 58.5% in L65 and change 83.8 to 83.8% in L67.

3. The meaning of some percentages such as “93.5% (pH: 7.0, 93.5%), 85.1% (pH: 8.0, 85.1%) and 70.5% (pH: 4.6, 70.5%) in L83 and 84 is not clear.

4. To make the meaning of the sentence of “Samples were shaken for 1 h on a reciprocal shaker, filtered and the extract analyzed on a Continuous Flow Analyzer “ clearer (i.e. the contents of NH4^+^-N and NO_3_^—^N in the extract of soil were analyzed on a Continuous Flow Analyzer), the two sentences “The soil NH4^+^-N and NO_3_^—^N content were determined by extracting a 5-g soil subsamples with 50 ml of 2 mol L^-1^ potassium chloride (KCl)” in L157 and “Samples were shaken for 1 h on a reciprocal shaker, filtered and the extract analyzed on a Continuous Flow Analyzer” in L158 should be combined into one sentence, for example, change the two sentences to “The soil NH4^+^-N and NO_3_^—^N content were determined by extracting a 5-g soil subsamples with 50 ml of 2 mol L^-1^ potassium chloride (KCl) and the samples were shaken for 1 h on a reciprocal shaker, filtered and the extract analyzed on a Continuous Flow Analyzer”.

5.L161：Is the concept “Apparent nitrification rate” in line 161 mentioned for the first time? If this concept is mentioned for the first time, the significance of calculating “Apparent nitrification rate” should be introduced briefly. Otherwise, the reader does not know why “Apparent nitrification rate” should be calculated in this paper.

6. The abbreviation “ANR” of “Apparent nitrification rate” should be clearly marked when it appears for the first time in L204. Moreover, what the reviewers don't understand is that the “Apparent nitrification rate” in the first sentence in L204 is abbreviated, but why does the “Apparent nitrification rate” in the second sentence in L204 and 205 use the full name?
