# Supplementary material for: Effects of combined nitrification inhibitors on soil nitrification, maize yield and nitrogen use efficiency in three agricultural soils
Source: PLoS One. 2022 Aug 22;17(8):e0272935. doi: 10.1371/journal.pone.0272935 (PMC9394818; doi:10.1371/journal.pone.0272935)
Supplement: Attachment — Submitted filename: Response to reviewers.docx [file pone.0272935.s005.docx]

Dear Manuel Joaquín Reigosa, Ph.D.,

Thank you very much for your letter and the reviewers’ reports concerning our manuscript entitled “PONE-D-21-09541”. Those comments are valuable and very helpful for revising and improving our paper. We have read through comments carefully and have made corrections. Based on the instructions provided in your letter, we uploaded the file of the revised manuscript. Revisions in the text are shown using red highlight for easy checking purpose. The responses to the reviewer's comments are presented following.

**Response to academic editor’s comments**

1. Please review your reference list to ensure that it is complete and correct. If you have cited papers that have been retracted, please include the rationale for doing so in the manuscript text, or remove these references and replace them with relevant current references. Any changes to the reference list should be mentioned in the rebuttal letter that accompanies your revised manuscript. If you need to cite a retracted article, indicate the article’s retracted status in the References list and also include a citation and full reference for the retraction notice.

We have reviewed the reference list to ensure that it is complete and correct.

**Response to reviewers’ comments**

**Reviewer #1:** I am happy to see that the authors have answered all the queries raised by the reviewers. After revision the MS is scientifically more sound than the first submitted form. In my opinion the MS is fit for publication.

RESPONSE: Thank you for your approval.

**Reviewer #2:** The manuscript has been carefully revised by the author, but there are still several small problems as follows：

RESPONSE: We appreciate the suggestions from the reviewer, which help us greatly improve our manuscript. The specific responses to the comments are as follows.

1. L26 and L102,103: The active voice such as “we conducted an outdoor pot ……” used in the abstract L26 is inconsistent with the passive voice such as “an outdoor pot experiment with different types of NIs additions in the above three soils was conducted” in the preface L102,103. It suggested that the passive voice should be used as much as possible in the full text.

We fully agreed with the suggestion of the reviewer. According to the suggestions, we described in the passive voice in the abstract L26.

2. L65，67: There are no percentage signs for some quantities such as “58.5 in L65” and “83.8” that need to be added with a percentage sign “%”, i.e. change 58.5 to 58.5% in L65 and change 83.8 to 83.8% in L67.

It was added.

3. The meaning of some percentages such as “93.5% (pH: 7.0, 93.5%), 85.1% (pH: 8.0, 85.1%) and 70.5% (pH: 4.6, 70.5%) in L83 and 84 is not clear.

We have rewritten the sentence to make the content clearer. The meaning of “93.5%”, “85.1%” and “70.5%” was the nitrification inhibition rate in the neutral soil (pH: 7.0), alkaline soil (pH: 8.0) and acid soil (pH: 4.5), respectively.

4. To make the meaning of the sentence of “Samples were shaken for 1 h on a reciprocal shaker, filtered and the extract analyzed on a Continuous Flow Analyzer “ clearer (i.e. the contents of NH_4_^+^-N and NO_3_^-^-N in the extract of soil were analyzed on a Continuous Flow Analyzer), the two sentences “The soil NH_4_^+^-N and NO_3_^-^-N content were determined by extracting a 5-g soil subsamples with 50 ml of 2 mol L-1 potassium chloride (KCl)” in L157 and “Samples were shaken for 1 h on a reciprocal shaker, filtered and the extract analyzed on a Continuous Flow Analyzer” in L158 should be combined into one sentence, for example, change the two sentences to “The soil NH_4_^+^-N and NO_3_^-^-N content were determined by extracting a 5-g soil subsamples with 50 ml of 2 mol L-1 potassium chloride (KCl) and the samples were shaken for 1 h on a reciprocal shaker, filtered and the extract analyzed on a Continuous Flow Analyzer”.

We fully agreed with the suggestion of the reviewer. We have combined the two sentences into one sentence.

5.L161：Is the concept “Apparent nitrification rate” in line 161 mentioned for the first time? If this concept is mentioned for the first time, the significance of calculating “Apparent nitrification rate” should be introduced briefly. Otherwise, the reader does not know why “Apparent nitrification rate” should be calculated in this paper.

We have introduced the significance of calculating “Apparent nitrification rate”briefly.

6. The abbreviation “ANR” of “Apparent nitrification rate” should be clearly marked when it appears for the first time in L204. Moreover, what the reviewers don't understand is that the “Apparent nitrification rate” in the first sentence in L204 is abbreviated, but why does the “Apparent nitrification rate” in the second sentence in L204 and 205 use the full name?

To make the contents clearer, we have changed the abbreviation “ANR” to “Apparent nitrification rate” when it appears for the first time. Moreover, the abbreviation “ANR” of “Apparent nitrification rate” was used in the second sentence.

**Reviewer #3:** This manuscript describes the effects of nitrification inhibitors on nitrification and maize yield in different agricultural soils, which is a very meaningful study. The authors have adequately addressed the comments raised in a previous round of review and this manuscript is now acceptable for publication. The manuscript has described a technically sound piece of scientific research with data that supports the conclusions, and the statistical analysis has been performed appropriately and rigorously. In my opinion, this work is well written and provides a theoretical basis for utilization. Therefore, I suggest that the article could be accepted and published in this journal.

RESPONSE: Thank you for your approval.

We would like to thank you for allowing us to submit a revised manuscript with minor revision and we highly appreciate your time and consideration.

Sincerely,

Professor Dongpo Li
