# Supplementary material for: Effects of combined nitrification inhibitors on soil nitrification, maize yield and nitrogen use efficiency in three agricultural soils
Source: PLoS One. 2022 Aug 22;17(8):e0272935. doi: 10.1371/journal.pone.0272935 (PMC9394818; doi:10.1371/journal.pone.0272935)
Supplement: Attachment — Submitted filename: PONE-D-21-09541_R3_reviewer.docx [file pone.0272935.s006.docx]

PONE-D-21-09541_R3_reviewer

Effects of combined nitrification inhibitors on soil nitrification, maize yield and nitrogen use efficiency in three agricultural soils

Comments on contents：

1. In this study, three soils with different pH were used for pot experiment, which showed that the biggest difference in physical and chemical properties of three different types of soils was pH. However, the conclusion showed that pH was the main factor affecting the utilization rate of three soil nitrification inhibitors without discussing the impact of other soil properties, this conclusion was preconceived and far fetched. Is the conclusion credible?

2. In Table 1, except for soil pH, other factors are the total amount and available content of nutrient elements affected by soil properties, which cannot be used as soil properties.

3. In “materials and methods”: soil sample collection methods are described in the lines of 105 and 139. What is the relationship or difference between them in L105 and 139? It is suggested to combine these two parts related to soil sample collection.

4. L345 and 346：As one of conclusions “The study shows that the effect of different NIs varied among different soils, and soil pH is the main factor.” The conclusion is too far fetched, and the statement of the conclusion is too vague and the content is not clear.

5. L80，81: “nitrification is rapid in soils of pH ≥ 6.0, but slower in soils of pH ≤ 5.0”(Is there a quantitative calculation to express speed in terms of rate? It can be expressed as easy or not)

Comments on grammatical errors or nonstandard expressions:

1. L169: It suggested that the title “NH_4_ ^+^ -N content and NO _3_ ^-^ -N content” should be changed to “The contents of NH_4_ ^+^ -N and NO _3_ ^-^ -N in soils”

2. It is not necessary to emphasize this research or frequently express research results or opinions in the first person during the results and discussion, otherwise the subjectivity of scientific research papers is too strong and does not accord with the objectivity of scientific papers. For example：

L27: “ In the present research”

L75: “Our study therefore addresses”

L170：“We clearly observed“

L179: “We also found that”

L294：“We also found that at the……”

L313: “which was in contrast to our results”

L320：“thus our best choice”,

L323 and 326：“In our study”

L332：“The results was similarly to our study”

L340: “In our study, we clearly observed”

L345：“The study shows that”(The conclusion is of course the conclusion of this study, and this emphasis has no significance at all)

3. The sentences in L47 and 48 should be combined into compound sentences(“asignificantly contribution in alleviating the global food shortage. Hence, tons of N fertilizers arepplied to obtain high yield of grain”)

4. Grammer irregularities or errors:

L59: “Two NIs, 3,4-dimethylpyrazol phosphate (DMPP) and dicyandiamide (DCD), which are commonly used” (There is no predicate, and the clause guided by which is redundan)

L77: “Different NIs have different effects mainly due to the properties of NIs,”(What is the impact, Unclear content description)

L310: “Ammonium ions usually adsorb by clay particles or soil organic matter, thus decreasing N loss”(It's the passive voice, not the active voice)

L312: “However, the results of Huérfano et al and Guardia et al showed that there are no any effect of“ (The present tense should be the past tense)

L320-321: “thus our best choice is the application of N-GD and DMPP+N-GD in brown soil and cinnamon soil, respectively.”

L330: “the effectiveness of NIs also influenced by SOM”

5. Nonstandard expressions：

L64，66，67: “58.5 and 35.2%” , “0.3-41.1% a” , “6.3-34.4%” should be changed into “58.5% and 35.2%” , “0.3%-41.1% a” , “6.3%-34.4%”.

6. Unclear or confused logical relationship:

L87 and 88：“but in grain yield significantly higher than those of control treatment,”“ however, there was no significant difference between NIs and without NIs [22]. But another field……”(but……, however……… but….. How to understand these three continuous turning relationships? )

L281 and 282：It is worth to mention that nitrification in red soil becomes stronger, which was according with Lu et al [31], mainly due to the tillage during the decades(How to understand the relationship between “which was according with……., mainly due to ” ).
