# Supplementary material for: Effects of combined nitrification inhibitors on soil nitrification, maize yield and nitrogen use efficiency in three agricultural soils
Source: PLoS One. 2022 Aug 22;17(8):e0272935. doi: 10.1371/journal.pone.0272935 (PMC9394818; doi:10.1371/journal.pone.0272935)
Supplement: Attachment — Submitted filename: Response to reviewers.docx [file pone.0272935.s008.docx]

Dear Manuel Joaquín Reigosa, Ph.D.,

Thank you very much for your letter and the reviewers’ reports concerning our manuscript entitled “PONE-D-21-09541”. Those comments are valuable and very helpful for revising and improving our paper. We have read through comments carefully and have made corrections. Based on the instructions provided in your letter, we uploaded the file of the revised manuscript. Revisions in the text are shown using red highlight for easy checking purpose. The responses to the reviewer's comments are presented following.

**Response to academic editor’s comments**

1. Please review your reference list to ensure that it is complete and correct. If you have cited papers that have been retracted, please include the rationale for doing so in the manuscript text, or remove these references and replace them with relevant current references. Any changes to the reference list should be mentioned in the rebuttal letter that accompanies your revised manuscript. If you need to cite a retracted article, indicate the article’s retracted status in the References list and also include a citation and full reference for the retraction notice.

We have reviewed the reference list to ensure that it is complete and correct.

**Response to reviewers’ comments**

**Reviewer #1:** The authors have made detailed modifications according to the comments of reviewers, and I think it is now suitable for publication in this journal.

RESPONSE: Thank you for your approval.

**Reviewer #2:**

1. In this study, three soils with different pH were used for pot experiment, which showed that the biggest difference in physical and chemical properties of three different types of soils was pH. However, the conclusion showed that pH was the main factor affecting the utilization rate of three soil nitrification inhibitors without discussing the impact of other soil properties, this conclusion was preconceived and far fetched. Is the conclusion credible?

Thank you for your review. We have rewritten the conclusion to make it credible. In the discussion part, except for soil pH, we also have discussed the impact of soil organic matter (SOM) on the efficiency of nitrification inhibitors. Nitrification inhibitors are compounds that delay the process of nitrification of NH_4_^+^ to NO_3_^-^. Therefore, Pearson correlation analysis between soil pH, soil organic matter (SOM), NH_4_^+^-N and NO_3_^-^-N was used to further confirmed our conclusion.

2. In Table 1, except for soil pH, other factors are the total amount and available content of nutrient elements affected by soil properties, which cannot be used as soil properties.

Thank you for your review. Generally speaking, the soil chemical properties refer to the chemical composition of soil, including the content of various elements in the soil. In addition, many literatures consider the content of nutrient elements in soil as soil properties.

3. In “materials and methods”: soil sample collection methods are described in the lines of 105 and 139. What is the relationship or difference between them in L105 and 139? It is suggested to combine these two parts related to soil sample collection.

Thank you for your review. Line 105 describes the tested soil in this study, while L139 is the collection method of soil samples for analysis in each growth period of pot experiment. We have changed “Study site and soil sampling” in L105 to “Study site and soils” to more clearly distinguish between them.

4. L345 and 346：As one of conclusions “The study shows that the effect of different NIs varied among different soils, and soil pH is the main factor.” The conclusion is too far fetched, and the statement of the conclusion is too vague and the content is not clear.

Thank you for your review. We have rewritten the conclusion“soil pH and SOM are the main factors affecting the efficiency of NIs”to make the content clearer. Moreover, we have discussed the impact of soil pH and soil organic matter (SOM) on the efficiency of nitrification inhibitors to make the conclusion credible.

5. L80，81: “nitrification is rapid in soils of pH ≥ 6.0, but slower in soils of pH ≤ 5.0”(Is there a quantitative calculation to express speed in terms of rate? It can be expressed as easy or not)

Thank you for your review. This is a general conclusion based on the literature cited, so there is not quantitative calculation to express speed in terms of rate. We have changed the expressed from “nitrification is rapid in soils of pH ≥ 6.0, but slower in soils of pH ≤ 5.0” to “nitrification is easy in soils of pH ≥ 6.0, but not in soils of pH ≤ 5.0”.

6. L169: It suggested that the title “NH_4_^+^-N content and NO_3_^-^-N content” should be changed to “The contents of NH_4_^+^-N and NO_3_^-^-N in soils”

Thank you for your review. We have changed.

7. It is not necessary to emphasize this research or frequently express research results or opinions in the first person during the results and discussion, otherwise the subjectivity of scientific research papers is too strong and does not accord with the objectivity of scientific papers. For example：

L27: “ In the present research”

L75: “Our study therefore addresses”

L170: “We clearly observed“

L179: “We also found that”

L294：“We also found that at the……”

L313: “which was in contrast to our results”

L320：“thus our best choice”,

L323 and 326：“In our study”

L332：“The results was similarly to our study”

L340: “In our study, we clearly observed”

L345：“The study shows that”(The conclusion is of course the conclusion of this study, and this emphasis has no significance at all)

Thank you for your review. We fully agreed with the suggestion of the reviewer. We have deleted them and expressed research results or opinions in the third person during the results and discussion.

8. The sentences in L47 and 48 should be combined into compound sentences(“a significantly contribution in alleviating the global food shortage. Hence, tons of N fertilizers are applied to obtain high yield of grain”)

Thank you for your review. We have combined the sentences in L47 and L48 into compound sentences (“Hence, tons of N fertilizers are applied to obtain high yield of grain, which made a significant contribution in alleviating the global food shortage”).

9. Grammer irregularities or errors:

L59: “Two NIs, 3,4-dimethylpyrazol phosphate (DMPP) and dicyandiamide (DCD), which are commonly used” (There is no predicate, and the clause guided by which is redundan)

L77: “Different NIs have different effects mainly due to the properties of NIs,”(What is the impact, Unclear content description)

L310: “Ammonium ions usually adsorb by clay particles or soil organic matter, thus decreasing N loss”(It's the passive voice, not the active voice)

L312: “However, the results of Huérfano et al and Guardia et al showed that there are no any effect of“ (The present tense should be the past tense)

L320-321: “thus our best choice is the application of N-GD and DMPP+N-GD in brown soil and cinnamon soil, respectively.”

L330: “the effectiveness of NIs also influenced by SOM”

Thank you for your review. L59: It was changed to “Two commonly used NIs are 3,4-dimethylpyrazol phosphate (DMPP) and dicyandiamide (DCD)”.

L77: It was changed to “The main factors affecting the effect of NIs are the properties of NIs,” to make the content clearer.

L310: It was changed the passive voice.

L312: It was changed

L320-321: It was changed to “thus the best choice in the brown soil and cinnamon soil is the application of N-GD and DMPP+N-GD, respectively.” to make the content clearer.

L330: It was changed to “SOM also affected the effectiveness of NIs”.

10. Nonstandard expressions：

L64，66，67: “58.5 and 35.2%” , “0.3-41.1% a” , “6.3-34.4%” should be changed into “58.5% and 35.2%” , “0.3%-41.1% a” , “6.3%-34.4%”.

Thank you for your review. It was changed in the revised manuscript.

11. Unclear or confused logical relationship:

L87 and 88：“but in grain yield significantly higher than those of control treatment,”“ however, there was no significant difference between NIs and without NIs [22]. But another field……”(but……, however……… but….. How to understand these three continuous turning relationships? )

L281 and 282：It is worth to mention that nitrification in red soil becomes stronger, which was according with Lu et al [31], mainly due to the tillage during the decades(How to understand the relationship between “which was according with……., mainly due to ” ).

Thank you for your review. L87 and L88: It was changed in the revised manuscript to make the content clearer.

L281 and 282: To make the logical relationship clearer, it was changed to “It is worth to mention that nitrification in the red soil has become stronger after tillage in recent decades, which is according with Lu et al”.

We would like to thank you for allowing us to submit a revised manuscript and we highly appreciate your time and consideration.

Sincerely,

Professor Dongpo Li
