# Supplementary material for: Effects of combined nitrification inhibitors on soil nitrification, maize yield and nitrogen use efficiency in three agricultural soils
Source: PLoS One. 2022 Aug 22;17(8):e0272935. doi: 10.1371/journal.pone.0272935 (PMC9394818; doi:10.1371/journal.pone.0272935)
Supplement: Attachment — Submitted filename: Effects of combined nitrification inhibitors on soil nitrification-reviwer comments.docx [file pone.0272935.s009.docx]

Effects of combined nitrification inhibitors on soil nitrification, maize yield and nitrogen use efficiency in three agricultural soils

Comments:

The author has carefully revised and improved the manuscript according to the reviewers' comments, but there are still the following problems:

1. Pay attention to the correct use of tense: the results of the cited literature and the research results of this paper should be in the past tense.

2. It is recommended to use a three line table instead.

3. The font size of abscissa, ordinate and legend of all figures are too small to identify their meaning.

4. Abbreviations should not be used in conclusions, it is suggested that the abbreviations in the conclusion should be expressed in full to make the conclusion more clear and definite.
