# Supplementary material for: Effects of combined nitrification inhibitors on soil nitrification, maize yield and nitrogen use efficiency in three agricultural soils
Source: PLoS One. 2022 Aug 22;17(8):e0272935. doi: 10.1371/journal.pone.0272935 (PMC9394818; doi:10.1371/journal.pone.0272935)
Supplement: Attachment — Submitted filename: Response to reviewers.docx [file pone.0272935.s010.docx]

Dear Rafiq Islam, Ph.D.,

Thank you very much for your letter and the reviewers’ reports concerning our manuscript entitled “PONE-D-21-09541”. Those comments are valuable and very helpful for revising and improving our paper. We have read through comments carefully and have made corrections. Based on the instructions provided in your letter, we uploaded the file of the revised manuscript. Revisions in the text are shown using red highlight for easy checking purpose. The responses to the reviewer's comments are presented following.

**Response to additional editor’s comments**

1．The abstract of the manuscript needs more focus especially on actual results, significant digits, and experimental design. Likewise, introduction should have appropriate citations, avoid superlative words, and add a hypothesis. Soils information presented in the introduction should go under study sites in M&M section.

Thank you for your review. We have revised abstract and added a hypothesis in the introduction. Additionally, soils information presented in the introduction have been placed in M&M section.

3. In M&M section, soils nomenclature should be used properly. There is a lack of information on experimental design and analytical methodologies. The statistical analysis of data is incomplete when data were measured over time. There should be an inclusion of time dependent random factor for few measurements. Some data need to be analyzed by using 2-way analysis of variance, while data measured over time need to be analyzed by using 3-way analysis of variance for treatment, soil, time, treatment x soil, treatment x time, soil x time, and treatment x soil x time effects. Needs to reanalyzed the data.

Thank you for your review. We have added the information on experimental design and analytical methodologies and analyzed the data measured over time by using 3-way analysis of variance.

4. Furthermore, how was soil moisture adjusted at 60% throughout the pot? How the soil water holding capacity was measured? Needs to explain the methodology.

Thank you for your review. The method of measuring soil water holding capacity is as follows: Firstly, take soil with a ring knife, bring it back to the laboratory, lay a filter paper under the ring knife, fix it on the ring knife with rubber band; secondly, put the ring knife on a plate, pour water into the plate until the filter paper is soaked, the soil in the ring knife was removed and weighed in the aluminum box of known weight to obtain the soil mass W1 at the maximum water holding capacity; thirdly, the aluminum box was dried in the oven at 105℃ for more than 8 hours to constant weight, and then weighed to obtain the dry soil mass W2; finally, the maximum water holding capacity was obtained according to the formula (W1-W2) /W2. The soil water content adjusted about 60% of the maximum water holding capacity in the field, that is, the soil water content was about 20%. The specific steps are as follows: 1) The water content of the tested soil is determined as A (%); 2) When the water content is A, the weight of the tested soil required for 8kg dry soil (the amount of soil in each pot is converted to 8kg dry soil) is calculated，which is B (kg); 3) When the water content is 20%, the weight of the tested soil required for 8kg dry soil, namely C (kg); 3) B-C refers to the weight of water added to the soil, so that the water content in each pot of soil reaches about 60% of the maximum water holding capacity in the field.

5. Results and discussion will change as per new statistical analysis. Correlation presented in Table 3 is linear; but it needs to check whether are there any non-linear responses.

Thank you for your review. We have changed the results and discussion. Additionally, we have made a linear regression, the results of linear regression are shown below, and their significance are 0.000, so there are no none-linear responses.

6. Graphs ( 1 and 2) were wrong. If growthstages are used as a qualitative predictor variable, then graphs should be prepared in bars. Line graphs for continuous variables like days (number). The authors should use days after planting (DAP) as a quantitative variable to prepare line graphs.

Thank you for your review. We have used days after planting (DAP) as a quantitative variable to prepare line graphs.

**Response to reviewers’ comments**

**Reviewer #1:** The authors have addressed all my comments. The quality of the paper has been improved, thus I have no further comments.

RESPONSE: Thank you for your approval.

**Reviewer #2:**

1. Pay attention to the correct use of tense: the results of the cited literature and the research results of this paper should be in the past tense.

Thank you for your review. We have corrected the use of tense.

2. It is recommended to use a three line table instead.

Thank you for your review. But in order to meet the journal's Table guidelines, we used the current table.

3. The font size of abscissa, ordinate and legend of all figures are too small to identify their meaning.

Thank you for your review. We have adjusted the font size of abscissa, ordinate and legend of all figures to identify their meaning.

4. Abbreviations should not be used in conclusions, it is suggested that the abbreviations in the conclusion should be expressed in full to make the conclusion more clear and definite.

Thank you for your review. We have expressed the abbreviations in full to make the conclusion more clear and definite.

**Reviewer #3:** The authors have solved the reviewers' concerns. I have no futher comments. I recommend its accpetance for publication in this journal.

RESPONSE: Thank you for your approval.

We would like to thank you for allowing us to submit a revised manuscript and we highly appreciate your time and consideration.

Sincerely,

Professor Dongpo Li
